# Electrical performance of efficient quad-crescent-shaped Si nanowire solar cell

**DOI:** 10.1038/s41598-021-03597-x

**Published:** 2022-01-07

**Authors:** Ramy El-Bashar, Mohamed Hussein, Salem F. Hegazy, Yehia Badr, B. M. A. Rahman, Kenneth T. V. Grattan, Mohamed Farhat. O. Hameed, Salah S. A. Obayya

**Affiliations:** 1 National Institute of Laser Enhanced Sciences (NILES), Cairo University, Giza, 12613 Egypt; 2 Centre for Photonics and Smart Materials, Zewail City of Science and Technology, October Gardens, 6th of October City, 12578 Giza Egypt; 3 Light Technology Institute, Karlsruhe Institute of Technology, Engesserstrasse 13, Karlsruhe, 76131 Germany; 4 Department of Physics, Faculty of Science, Ain Shams University, Abbassia, 11566 Cairo Egypt; 5 Department of Electrical and Electronic Engineering, City, University of London, London, EC 1 V 0HB UK; 6 Nanotechnology and Nanoelectronics Engineering Program, Zewail City of Science and Technology, October Gardens, 6th of October City, 12578 Giza Egypt; 7 Mathematics and Engineering Physics Department, Faculty of Engineering, University of Mansoura, Mansoura, 35516 Egypt; 8 Department of Electronics and Communication Engineering, Faculty of Engineering, University of Mansoura, Mansoura, 35516 Egypt

**Keywords:** Energy science and technology, Optics and photonics

## Abstract

The electrical characteristics of quad-crescent-shaped silicon nanowire (NW) solar cells (SCs) are numerically analyzed and as a result their performance optimized. The structure discussed consists of four crescents, forming a cavity that permits multiple light scattering with high trapping between the NWs. Additionally, new modes strongly coupled to the incident light are generated along the NWs. As a result, the optical absorption has been increased over a large portion of light wavelengths and hence the power conversion efficiency (PCE) has been improved. The electron–hole (e–h) generation rate in the design reported has been calculated using the 3D finite difference time domain method. Further, the electrical performance of the SC reported has been investigated through the finite element method, using the Lumerical charge software package. In this investigation, the axial and core–shell junctions were analyzed looking at the reported crescent and, as well, conventional NW designs. Additionally, the doping concentration and NW-junction position were studied in this design proposed, as well as the carrier-recombination-and-lifetime effects. This study has revealed that the high back surface field layer used improves the conversion efficiency by $\sim$ 80%. Moreover, conserving the NW radial shell as a low thickness layer can efficiently reduce the NW sidewall recombination effect. The PCE and short circuit current were determined to be equal to 18.5% and 33.8 mA$/\hbox {cm}^2$ for the axial junction proposed. However, the core–shell junction shows figures of 19% and 34.9 mA$/\hbox {cm}^2$. The suggested crescent design offers an enhancement of 23% compared to the conventional NW, for both junctions. For a practical surface recombination velocity of $10^{2}$ cm/s, the PCE of the proposed design, in the axial junction, has been reduced to 16.6%, with a reduction of 11%. However, the core–shell junction achieves PCE of 18.7%, with a slight reduction of 1.6%. Therefore, the optoelectronic performance of the core–shell junction was marginally affected by the NW surface recombination, compared to the axial junction.

## Introduction

Today, energy based on ‘green’ resources has attracted considerable interest and investment worldwide, as a viable alternative to the use of polluting fossil fuels. Solar energy is the most abundant source of such ‘green’ renewable energy, coming in its two forms: light and heat. The photovoltaic (PV) solar cell (SC) is one of the predominant solar energy harvesting devices available today; one that can be used effectively to convert light directly to electricity^1^. Currently, bulk crystalline silicon (C-Si) photovoltaic modules have $\sim$ 90% of the global PV market. Silicon (Si), as a material, is known as one of the largest broadband light absorbing materials, where the power conversion efficiency (PCE) of a planar C-Si SC reaches 22%^2^. However, the downside is that the thickness and the crystalline quality needed increase the price of the SC^3^. Second generation SC technology, based on low-purity thin-film (TF) materials, has emerged to reduce the high cost of the traditional C-Si SC and yet, the conversion efficiency is only $\sim$ 12%, due to the small optical path length seen, especially for low photon energies^4^. Besides that, low purity materials have a higher level of defects that reduce the diffusion length of the charge carriers, with increased recombination rate. The third generation of solar cell technology has improved the light absorption in Thin Films (TFs) using light trapping techniques. This approach leads to increasing path lengths and promotes the generation of e–h carriers, which elevates the efficiency of such TF SC. Consequently, an efficient TF SC can be designed using less active material and hence the whole device is available at a lower cost. Several structures utilizing this approach have been suggested, such as dielectric^5,6^, surface-patterned^7,8^, and plasmonic^9^ nanostructures, all designed to improve the light scattering, absorption and so the device PCE. It has been reported, further, that the whispering gallery modes of the dielectric nanospheres can be coupled with the SC modes^5^ and it has been found that the efficiency can be improved by enhancing the free space light coupling with the active layer, through excitation of new modes in the nanosphere^6^. Nanodome surface-patterning of a-Si:H with periodic modulation improves the light absorption and offers a higher short circuit current ($J_{sc}$) and PCE of 17.5 mA$/\hbox {cm}^2$ and 5.9%, respectively^7^. Wang and Leu^10^ have investigated such a double-sided nanocone design, which allows an improvement in the value of $J_{sc}$ to 34.6 mA$/\hbox {cm}^2$. Furthermore, integrating metallic nanostructures within the SC can allow the excitation of two forms of plasmons; localized plasmons at the nanoparticle surface^11^ or/and propagating plasmon polaritons at the interface of the semiconductor-metal back reflector^12^. Plasmonic nanostructures can obviously decrease the SC active layer, with an improved optical path^9^.

Nanowires (NW) are highly promising nanostructures that have unique optical and electrical characteristics compared to TF SCs^13^. Such NWs have a number of merits, such as reduction in reflection, improvement in trapping, and consumption of less material^13–15^ and several studies have reported improved efficiency of NW-based arrangements. Hu et al. have studied the effect of the filling ratio (FR) and the length of solid NWs on the light absorption^13^. In 2009, the effect of the nanostructure periodicity was investigated^16^ where the solid NW FR of 0.64, at 600 nm periodicity, was shown to have a maximum optical efficiency of 28%^16^. Also, lattice periodicities such as hexagonal and decagonal geometries, which show an enhancement in the light absorption, have been studied. Moreover, different types of SCs based on nanorods^13^, nanoholes^17^, nano-cones^10^ and nanopyramids^18^ and thus improving absorption. Therefore, the generated e–h carriers are increased with a positive effect of the PCE. Kordrostami and Yadallahi have also studied and compared the absorption behavior of various geometric nanostructure shapes^19^. The inverted-funnel and conical NWs investigated provide the highest light trapping and absorption, allowing PCE values of 18.23% and 18.15%, respectively to be achieved. Further, NW-based SCs offer higher carrier collection in the lported by Kayes et al.^20^ where a physical comparison between planar and radial-NW SC junctions was proposed. Their model presented emphasized that the radial type offers a higher PCE, relative to the planar SC. Moreover, the substrate, axial, and core–shell junctions have been reviewed, as well as the basics behind the PCE improvements seen^21,22^. It has been found that the core–shell p/n junction achieves a higher light path length and a smaller e–h carrier path length. Consequently, it offers a much better carrier extraction compared to the axial and substrate junctions. Additionally, hydrogenated amorphous silicon nanocone can be utilized to improve the SC conversion efficiency^23^. Adachi et al.^24^ have examined the doping of the shell layer with nanocrystalline (nc-Si) and amorphous (a-Si) silicon. The values of $J_{sc}$ and PCE of the nc-Si doping were found to be equal to 14.9 mA$/\hbox {cm}^2$ and 2.9%, respectively. However, values of 13.9 mA$/\hbox {cm}^2$ and 6% are seen for the a-Si shell doping. Furthermore, an asymmetric Si NW design has been proposed, showing a PCE of 7.4%^25^. By contrast, the PCE of SCs-based NWs may decrease due to an increased recombination over the high sidewall surface area^20^. To solve this problem, surface passivation techniques were employed for thin Si SC^26,27^. In 2011, Kim et al.^28^ had studied micro-wire Si SC with a substrate based on a core–shell junction. The PCE value was raised from 7 to 11%, using the hybrid SC, by considering the passivation layer. A heavily-doped Boron back surface field (B-BSF) structure has been proposed, that offered a better field-effect passivation than the conventional Al-BSF^29,30^. Zhang et al.^31^ have designed a NH SC based on dual-diameter approach with a maximum PCE of 13.72%. Recently, the PCE of the funnel design in the axial and core–shell junctions were reported to be 12.7% and 14.1%, respectively^32^. Additionally, Deinega et al.^33^ had proposed a conical pore SiNW design, where a PCE of 17.5% by using standard good contact recombination was proposed. In 2020, a design based on NWs with a crescent nanohole was proposed, which offered a PCE of 17%, with an enhancement of 17% compared to the cylindrical solid NW design^34^.

In light of the above, in this paper, the optoelectronic characteristics of a modified quad-crescent (QCr) NW SC are presented. The optical representation of the NW design has been recently reported by El-Bashar et al.^35^. In this work, the electrical parameters were studied with a fully coupled optoelectronic model, by using a Lumerical charge software package, based on the finite element method^36^. The electrical J–V characteristics of the axial and core–shell junctions are investigated for the proposed QCr-NW. The key parameters of the reported design studied are the generation rate, the short circuit current density ($J_{sc}$), the open circuit voltage ($V_{oc}$), and the fill factor (FF). The power conversion efficiency (PCE) has been calculated by taking the electrical characteristics of active material into account. Also, the effect of the donor and acceptor doping concentrations (besides the NW-junction position on the performance of the proposed QCr-NW SC) are examined, taking into consideration all the recombination effects. Moreover, the influence of the carrier lifetime and the surface recombination were calculated for both of the junctions studied. The design using the axial junction which has been suggested gives a value of $J_{sc}$ of 33.8 mA$/\hbox {cm}^2$ and a PCE of 18.5%. The efficiency achieved surpasses that of the asymmetric NW studied, with a crescent nanohole^34^ with a volume reduction of 45%. Furthermore, the PCE of the NW suggested in the core–shell is equal to 19%, with an enhancement of 23% compared to the solid NW. The efficiency enhancement achieved is due to the high level of generated carriers, with multiple scattered light between the cavities created. Moreover, the close matching of lateral effective index along the proposed design improves the coupling with the scattered light. It is evident that the core–shell junction allows a higher PCE compared to the axial junction. This is due to the efficient extraction of the generated carriers in the radial direction, especially though the small path length of teeth regions. Further, the thin radial shell layer reduces the surface minority carriers and thus the surface recombination effect. The PCE of the core–shell junction is equal to 18.7% and 4.3% compared to 16.5% and 0.8% of the axial junction at surface recombination velocities of $10^{2}$ cm/s and $10^{6}$ cm/s, respectively. Therefore, the core–shell junction offers a better electrical performance compared to the axial junction, especially where there is poor surface passivation.

## Design considerations and numerical methods

**Figure 1:**
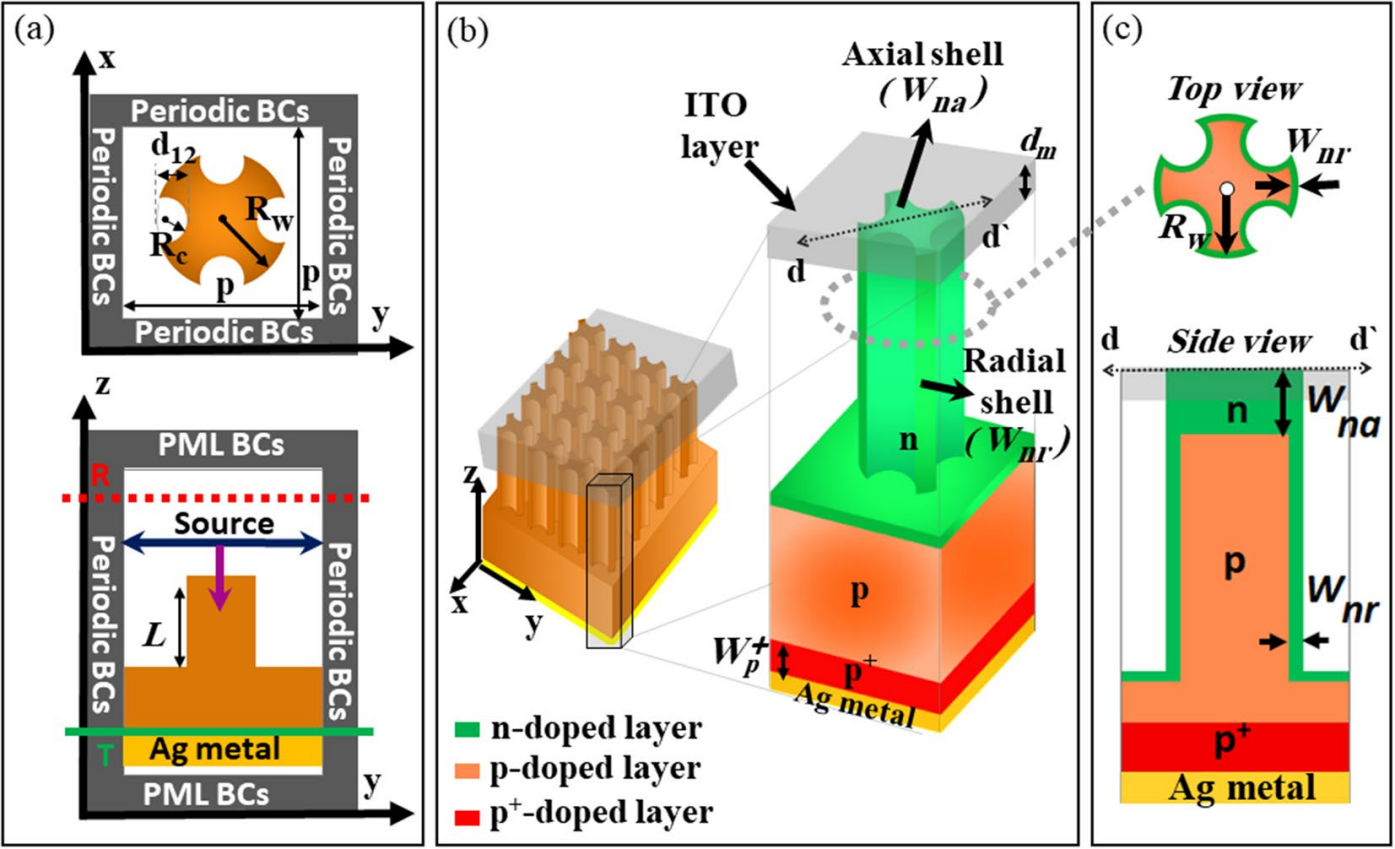
(**a**) Computational domain of the single unit cell in 2D plane. (**b**) 3D schematic diagram. (**c**) 2D top and side view of the proposed design in core–shell $n/p/p^+$ junction. The green region is n-doped, the orange region is p-doped, and the red region is the highly p-doped. This image is created by Lumerical 2020a, FDTD Solver Version 8.23.2305, https://www.lumerical.com (license number-12802) released to Zewail City of Science and Technology, Giza, Egypt.

In this study, the optoelectronic performance of a square array of QCr-NW has been studied. Figure 1 shows a schematic diagram of the QCr-NW design. The design proposed consists of a core cylinder with quad crescents. The core cylinder has a radius of $R_w$, while the crescent radius and width are $R_c$ and $d_{12}$, respectively and the distance between the centers of the core-cylinder and the crescent center is given by $D=R_{w}+R_{c}-d_{12}$. The QCr-NW proposed is set in a square array, over a 2000 nm thick Si substrate. The optical characteristics of the design suggested has been presented in recent work^35^ and Table 1 shows the geometrical parameters for the QCr-NW proposed. The optical characteristics of this QCr-NW have been studied by solving Maxwell’s equations, using the 3D FDTD method via the Lumerical software package^36^. A square single unit cell is taken with a periodic boundary layer in the x–y plane while a perfectly matched layer is used in ± z directions. To simulate the light source, a plane wave of AM-1.5G is taken, in the wavelength range from 300 to 1100 nm, with an average power density ($P_{in}$) of 100 mW$/\hbox {cm}^2$^37^. Figure 1a shows 2D computational domain for a single unit cell in the x–y and the x–z planes.

**Table 1:** The geometrical parameters values of the proposed and conventional solid NW structures^35^.

Parameters	Description	Con-NW	Proposed QCr-NW (nm)
L	NW length	2330 nm^51^	2330
$R_{w}$	Cylindrical NW radius	200 nm	200
$R_{c}$	Crescent radius	–	80^35^
$d_{12}$	Crescent width	–	100^35^
p	Lattice constant	600 nm^16^	600

**Figure 2:**
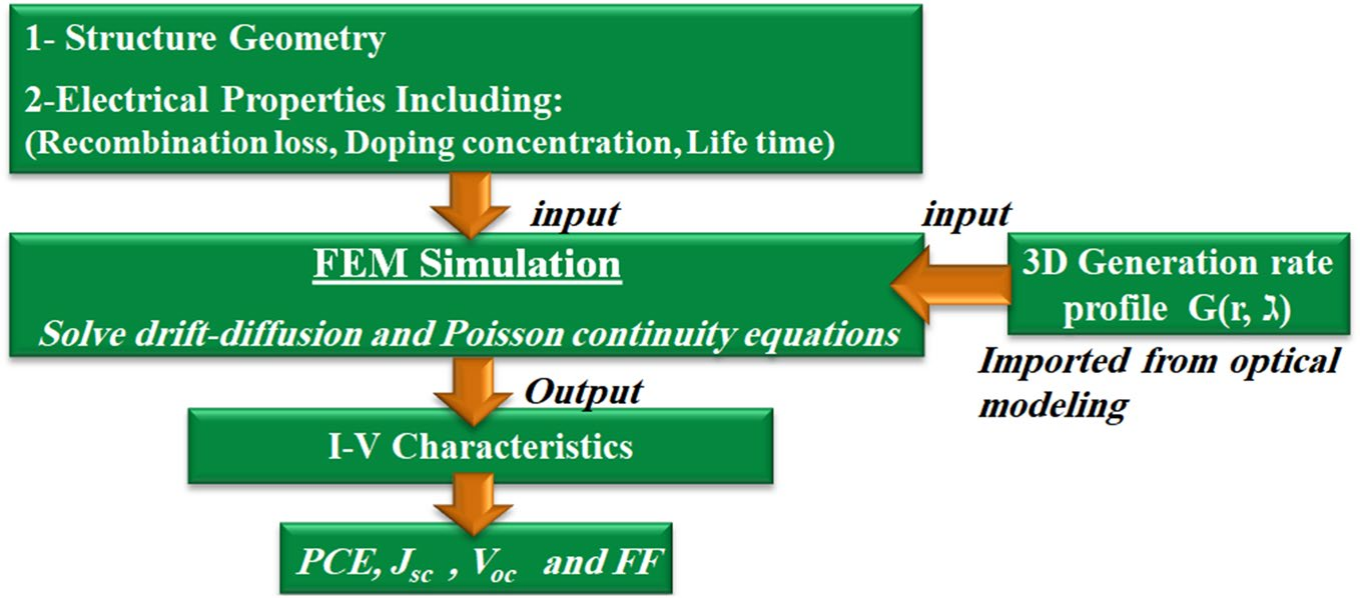
Flow chart of the electrical simulation strategy proposed.

To obtain the J–V characteristics under illumination, the optical generation profile is imported from the optical solver to the drift-diffusion equations of the charge solver. The 3D optical generation rate $G(r,\lambda )$ has been calculated from the position-dependent absorption spectra $(A(r,\lambda ))$ multiplied by the AM-1.5G photons spectra, which is given by^38^:

1 $$\begin{aligned} G(r,\lambda ) =A(r,\lambda ) ~I(\lambda ) ~\frac{\lambda }{hc}, \end{aligned}$$

where $\lambda$ is the wavelength, h is Planck’s constant,and $I(\lambda )$ is the solar irradiance of the AM-1.5G (global) irradiance^37^. The 3D $G(r,\lambda )$ is exported to the device continuity equation of the FEM based Lumerical charge solver. By calculating the coupled nonlinear drift and diffusion continuity, and Poisson equations, the carrier dynamics are obtained for both the studied designs^39^. To achieve realistic results, the losses related to radiative, trap-assisted Shockley-read-hall (SRH), Auger recombination, and the surface recombination are taken into account^40,41^. A flowchart illustrating the simulation steps is shown in Fig. 2. In this study, the surface recombination velocity (SRV) of the Si–Ag interface has been considered, as the maximum (saturation) velocity of carriers of $10^7$ cm/s^42,43^. To represent a practical good surface passivation, the SRV at Si-SiO2 interface is taken as $10^2$ cm/s^27^. The Si average carrier lifetime ($\tau _o$) is considered 3.3 $\upmu$s and 4 $\upmu$s, for electron and hole carriers, respectively^32^. Table 2 shows the electrical parameters of the Si active material. The maximum PCE is given by:

2 $$\begin{aligned} PCE = \frac{V_{p} J_{p}}{P_{in}}, \end{aligned}$$

where $V_{p}$ and $J_{p}$ are the operating load point voltage and current density, respectively, corresponding to the peak power point ($P_m$) of the SC. The fill factor (FF) is used to measure the IV-curve squareness and identify the maximum power conversion density of the SC, as given by^44^:

3 $$\begin{aligned} FF= \frac{V_{p} J_{p}}{V_{oc} J_{sc}}. \end{aligned}$$

In the optical study, a 3D computational domain of the unit cell was taken with a specific boundary condition to simulate the periodic arrangement^35^ of the studied designs. Subsequently, a periodic boundary condition (PBC) was inserted in x–y plane to minimize the computational time. Conversely, along the proposed design perfectly matched layers (PML) was utilized to diminish the reflections^16^ as shown in Fig. 1a.

Initially, the proposed design with the core–shell doping shown in Fig. 1b,c are studied and compared to the conventional cylindrical NW. In this investigation, the electrical characteristics of the QCr-NW proposed were obtained by using the finite element method via the Lumerical charge package^36^. The computational domain of the 3D charge solver is given by 600 nm, 600 nm and 4530 nm in the x, y and z directions, respectively. The back Ag electrode and the top contact of ITO material were taken as having a thickness of 200 nm^25,45^. Figure 1b,c show a schematic of the design proposed in the core–shell $n/p/p^+$-doped junctions, in 2D and 3D views. It has been shown that the minority carriers in the p-doped (i.e. electron) layer have higher mobility and lifetime than the n-doped (i.e. hole) layer^46,47^. Thus, the p-doped layer can be considered in this study for the NW core as the bottom substrate. Consequently, the (e–h) generated carriers in the thicker p-doped substrate layer have a higher probability of reaching the contacts. In this research, the dimension of the $p^+$-doped back-surface-field (BSF) ($W_p^+$) was taken as 400 nm^18,34^, which is smaller than the electron diffusion length thickness^48^. This layer helps in creating a barrier voltage for minority carrier recombination on the cathode Ag-metal contact^48^. The lateral n-doped radial ($W_{nr}$) shell and the axial top shell ($W_{na}$) were taken initially as 10 nm^49,50^. The p-doped core with substrate, and the $p^+$-doped BSF layers were initially taken as $1\times 10^{15}$ $\hbox {cm}^{-3}$, and $1\times 10^{19}$ $\hbox {cm}^{-3}$, respectively^48^, while the $n^+$-doping of shell layer was initially set to $1 \times 10^{18}$ $\hbox {cm}^{-3}$^49^. In this work, the emitter layer was used as n-type polycrystalline silicon, while the base layer was assumed to be p-type polycrystalline silicon. Table 2 shows also the geometrical widths and the initial doping concentrations of the QCr-NW design proposed in the core–shell junctions.

**Table 2:** The electrical and geometrical parameters of the proposed design in the core–shell junction.

Parameters	Description	Nominal value
$\mu _p$	Hole mobility	450 cm$^2/({\text{V}} \cdot {\text{s}})$^52,53^
$\mu _n$	Electron mobility	1400 cm$^2/({\text{V}} \cdot {\text{s}})$^52,53^
rad	Radiative recombination coefficient	$1.6 \times 10^{-14}$ cm$^{3}$/s^53^
n-Aug	Electron auger recombination	$2.8 \times 10^{-31}$ cm$^{6}$/s—300 K^54^
p-Aug	Hole auger recombination	$9.9 \times 10^{-32}$ cm$^{6}$/s—300 K^54^
SRV (Si–Ag)	Si Surface recombination velocity to Ag	$1 \times 10^{7}$ cm/s—300 K^43^
SRV (Si-$SiO_{2}$)	Si Surface recombination velocity to $SiO_{2}$	$1\times 10^{2}$ cm/s—300 K^27,33^
$\tau _o$	Si average carrier lifetime	$4 \times 10^{-6}$ s^55^
$W_{rs}$	n-doped radial (lateral) shell thickness	10 nm^49,50^
$W_{as}$	n-doped axial (top) shell thickness	10 nm
$d_m$	ITO layer thickness	200 nm^49,56^
$N_d$	n-type donor concentration	$1\times 10^{18}$ $\hbox {cm}^{-3}$^49,57^
$N_a$	p-type substrate acceptor concentration	$1 \times 10^{15}$ $\hbox {cm}^{-3}$^48^
$N_a^+$	$p^+$-type BSF concentration	$1 \times 10^{19}$ $\hbox {cm}^{-3}$^48^

## Results and discussion

**Figure 3:**
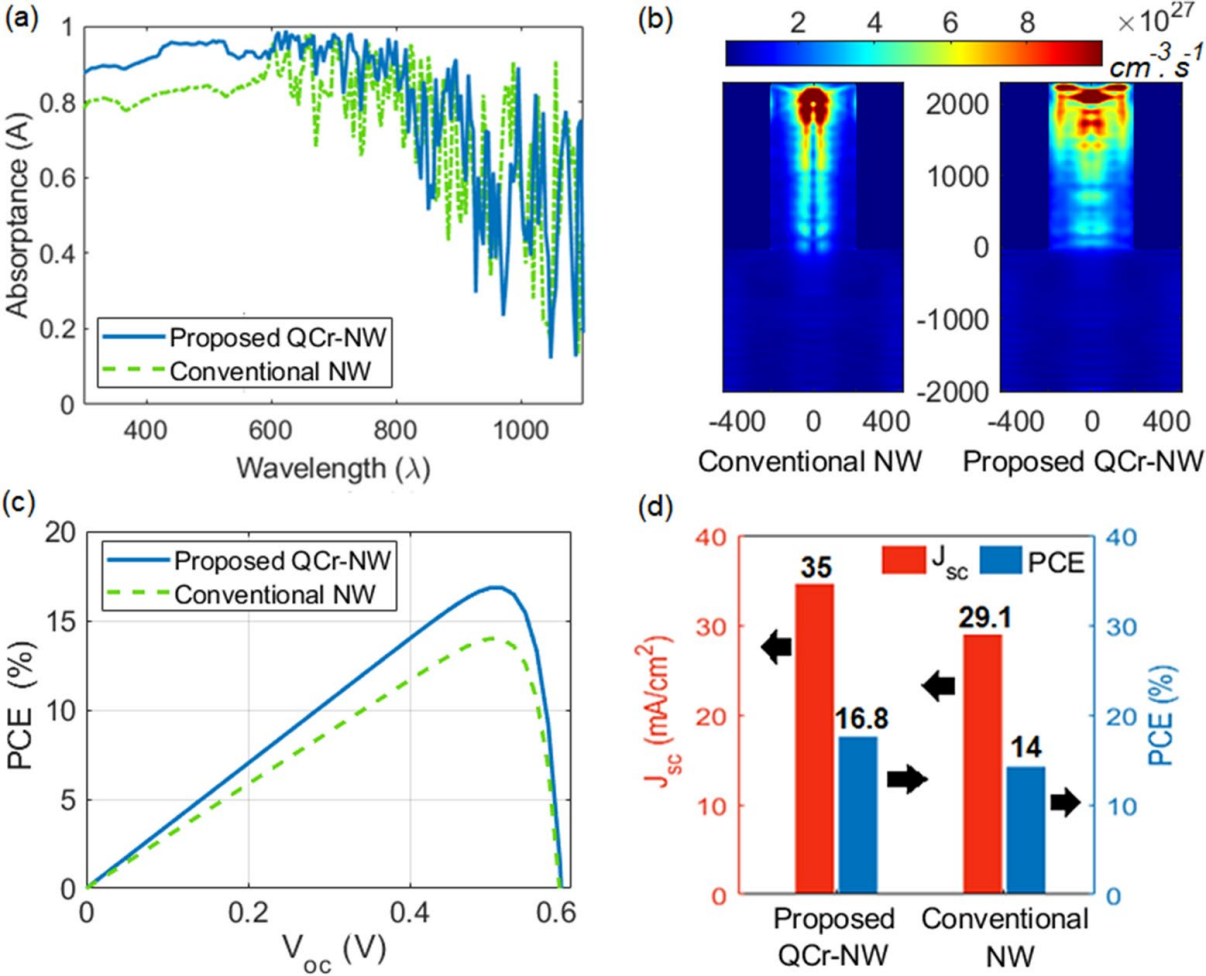
(**a**) Absorption spectra, (**b**) the optical generation rate profiles across the dd′ plane, (**c**) the PCE versus the $V_{oc}$ , and (**d**) the maximum PCE and $J_{sc}$, for the proposed crescent and conventional NW designs in the core–shell junction. This image is created by Lumerical 2020a, FDTD Solver Version 8.23.2305, https://www.lumerical.com (license number-12802) released to Zewail City of Science and Technology, Giza, Egypt.

Figure 3a shows the absorption spectra for both the proposed QCr and the conventional (Con-) NW designs. The geometrical parameters of the NW proposed is taken from Ref.^35^ as given in Table 1. Additionally, the conventional NW has a radius of 200 nm and a length of 2000 nm. It is evident from this figure that the reported design offers a higher light absorption, than does the cylindrical NW design. Therefore, the generation rate and hence the PCE are improved for the proposed design. Figure 3b shows the optical generation rate profile along the studied designs, across the dd′ plane for the both of these designs. To study the novel cavity created in the proposed design, the generation rate has been increased, compared to the conventional NW design. This cavity reduces the surface reflection and permits multiple light scattering with high trapping inside the NWs cavities. Moreover, the cavity geometry excites new modes and directs the light to the NW core to allow for superior absorption. The graphs showing the change in PCE versus the $V_{oc}$ of the reported core–shell of QCr-NW and the solid NW designs are presented in Fig. 3c. The maximum values of PCE and $J_{sc}$ are shown in Fig. 3d. It can be noticed that the QCr-NW suggested offers high $J_{sc}$ and PCE values compared with the solid NW counterpart. The maximum PCE of the QCr-NW proposed and the solid NW designs are 16.8% and 14%, respectively. Therefore, the QCr-NW proposed offers a higher PCE value, with an improvement of 20% compared to the conventional NW. Table 3 presents the data on the PCE for the initial core–shell junctions of the two designs studied.

**Figure 4:**
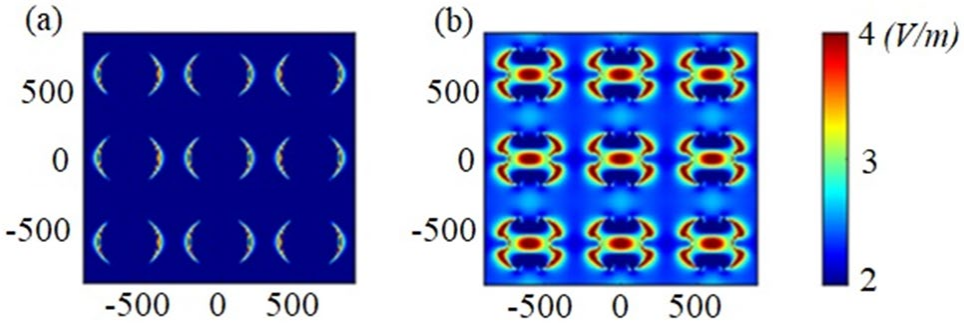
The field profiles for the (**a**) Con-NW and (**b**) QCr-NW designs at wavelength of 908 nm. This image is created by Lumerical 2020a, FDTD Solver Version 8.23.2305, https://www.lumerical.com (license number-12802) released to Zewail City of Science and Technology, Giza, Egypt.

It is evident that the incident light has further penetration and confinement via the proposed QCr-NW design. This backs to the increasing of the lateral surface area which strongly permits for multiple light scattering. Besides, the proposed design improves the total internal reflection inside each tooth compared to the conventional cylindrical NW. Therefore, the light is well-trapped not only in the core center but also expanded laterally to the graded teeth as shown in Fig. 3b. Figure 4a,b show the field profiles through $3\times 3$ periodic arrays of conventional and the proposed NW designs in the x–y plane at wavelength of 908 nm. It may be revealed that high Bloch modes^42,58^ are induced over the periodic QCr-NW design compared to the conventional NW design. Therefore, the optical efficiency of the proposed QCR-NW design is improved compared to the conventional cylindrical design.

**Table 3:** The PCE for the initial core–shell junctions of the QCr-NW and the conventional NW structures.

Parameters	Description	Core–shell junction	
		Proposed QCr-NW	Conventional solid NW
$J_{sc}$	Short circuit current density (mA$/\hbox {cm}^2$)	35	29.2
$V_{oc}$	Open circuit voltage (mV)	580	590
FF	Fill factor (%)	82	82
PCE	Power conversion efficiency	16.8	14

### Doping level effect

**Figure 5:**
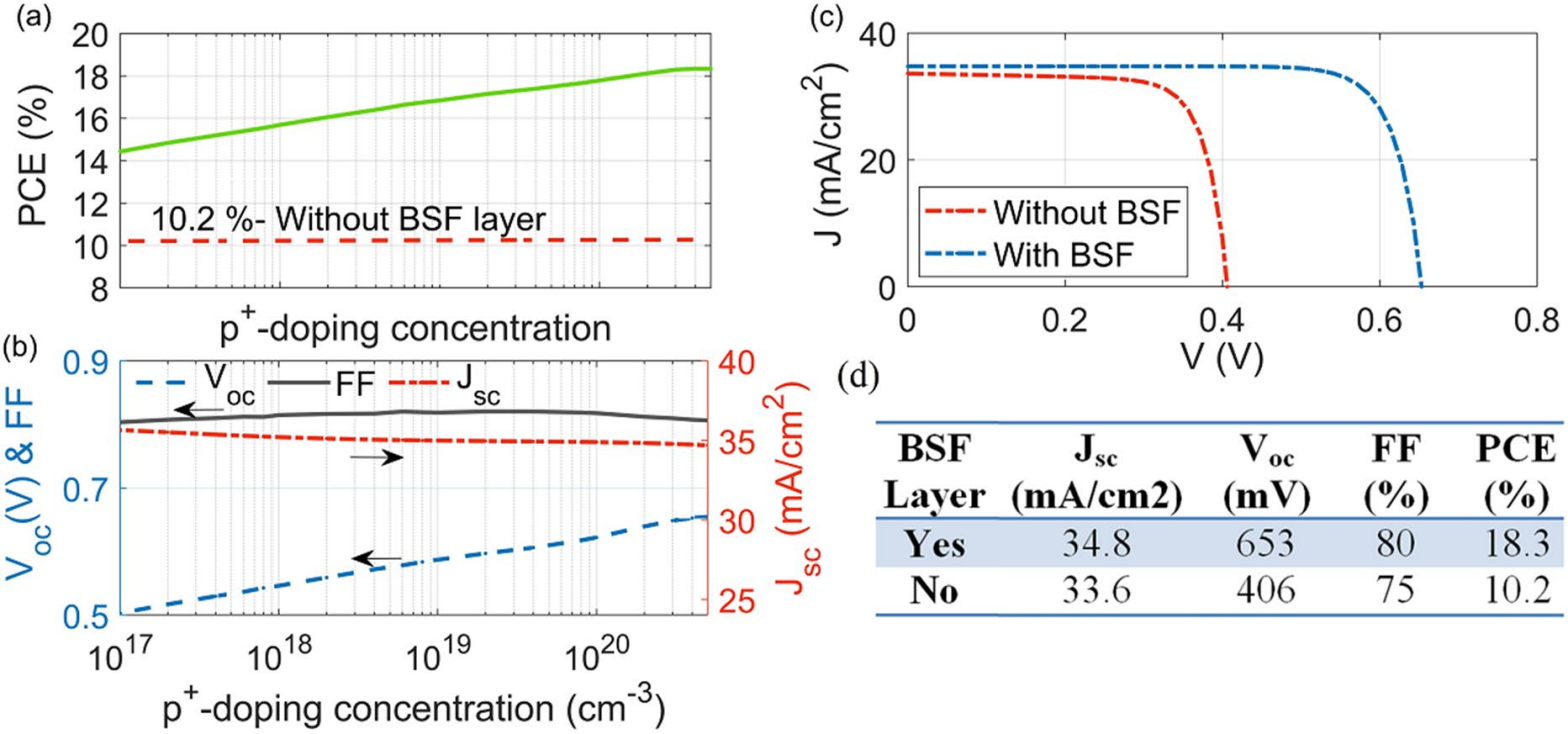
(**a**) PCE, (**b**) $V_{oc}$ , FF, and $J_{sc}$ versus the $p^+$-doped BSF layer, (**c**) J–V characteristics and (**d**) numerical calculations with and without using the optimized BSF layer ($Na^+=4\times 10^{20}$ $\hbox {cm}^{-3}$).

The doping levels required were first studied for the core–shell $n/p/p^+$ junction proposed. First, the $p^+$-doped level of the BSF layer has been optimized and Fig. 5a shows the change of the PCE (green line) versus the doping level of the $p^+$-doped layer ($\hbox {Na}^+$= $1 \times 10^{17}$ cm$^{-3}$ to $5\times 10^{20}$ cm$^{-3}$^59^). The values of the $V_{oc}$, FF, and the electrical $J_{sc}$ are presented in Fig. 5b. It can be seen that $J_{sc}$ is nearly constant, with the variation in the doping concentration, which may be due to the $p^+$-doped thickness which is smaller than the diffusion length^48^. However, the value of $V_{oc}$ is directly proportional to the BSF doping over the full range studied. This enhancement is due to the reverse field of the rear junction ($p/p^+$) created, that weakens the diffusion of the minority carriers (i.e. here electrons) to the rear surface^48^. The BSF layer density should be above 10$^{19}$ cm$^{-3}$, so that electron diffusion is minimized^48^. At a BSF value of $4 \times 10^{20}$ cm$^{-3}$ , the maximum PCE value of 18.3% was obtained at $V_{oc}$, $J_{sc}$ and FF of 653 mV, 34.7 mA$/\hbox {cm}^2$ and 0.81, respectively. Figure 5a shows also the performance of the QCr-NW proposed, in the absence of the BSF layer (dashed red line). To investigate the effect of the BSF layer, the J–V characteristics with and without the layer are shown in Fig. 5c. It can be seen that the PCE value has dropped to 10.2% (without the BSF). This confirms the efficiency enhancement of more than 79.5%, when the BSF layer was included. Figure 5d shows the calculated electrical output parameters of the proposed design with and without using the BSF layer.

**Figure 6:**
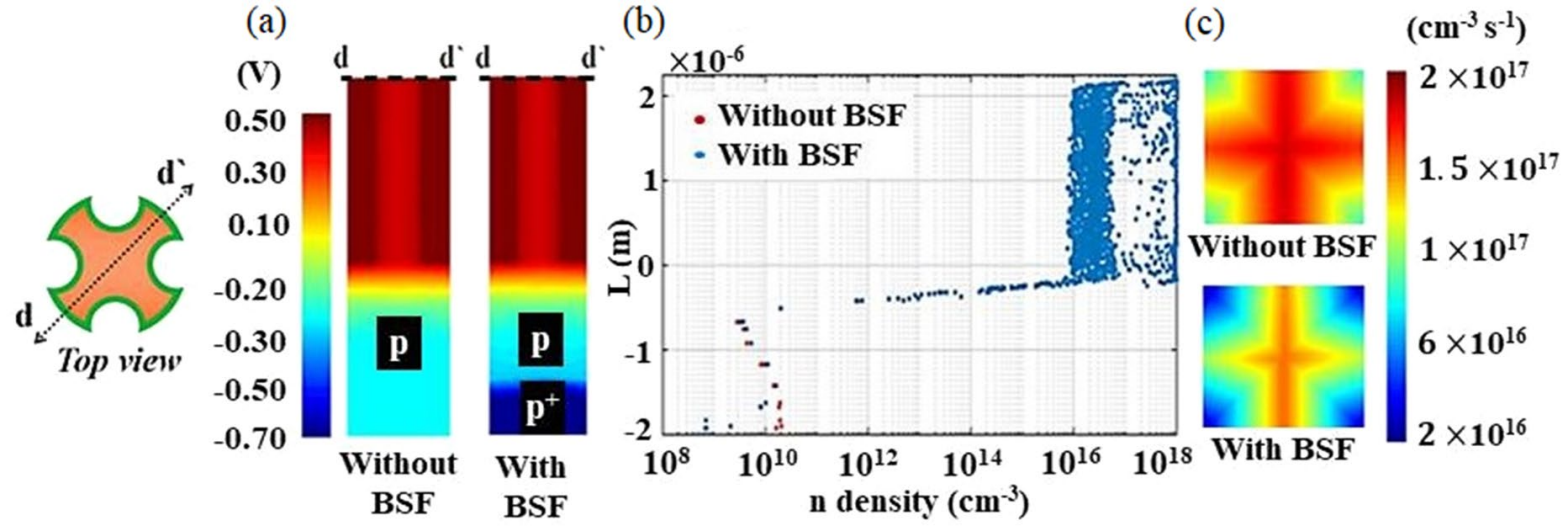
(**a**) Electric potential profile, (**b**) n-carrier concentration density, and SR rate (**c**) at the back surface metal layer, and with (right) and without (left) using the optimized BSF layer. This image is created by Lumerical 2020a, FDTD Solver Version 8.23.2305, https://www.lumerical.com (license number-12802) released to Zewail City of Science and Technology, Giza, Egypt.

Figure 6a depicts the electric voltage through the vertical $d{-}d^{\prime}$ plane, with and without using the BSF layer. It shows that the BSF layer could produce 300 mV in the reverse back voltage (the right one) at the bottom $p/p^+$ substrate junction. Figure 6b shows the electron minority carrier density with and without the BSF layer and it can be concluded that the increase of the $V_{oc}$ caused by the back $p/p^+$ junction is not related only to the increase of the BSF layer doping. The degradation of the minority carriers (i.e. electrons) at the $p^+$ doped back layer (shown by the  blue color) also contributes to the $V_{oc}$ enhancement^57^. Additionally, the surface recombination (SR) rate on the back metal contact, with and without using the BSF layer, is shown in Fig. 6c. It is evident that by including the $p^+$ doped BSF layer, the SR over the rear metal contact is seen to decrease.

**Figure 7:**
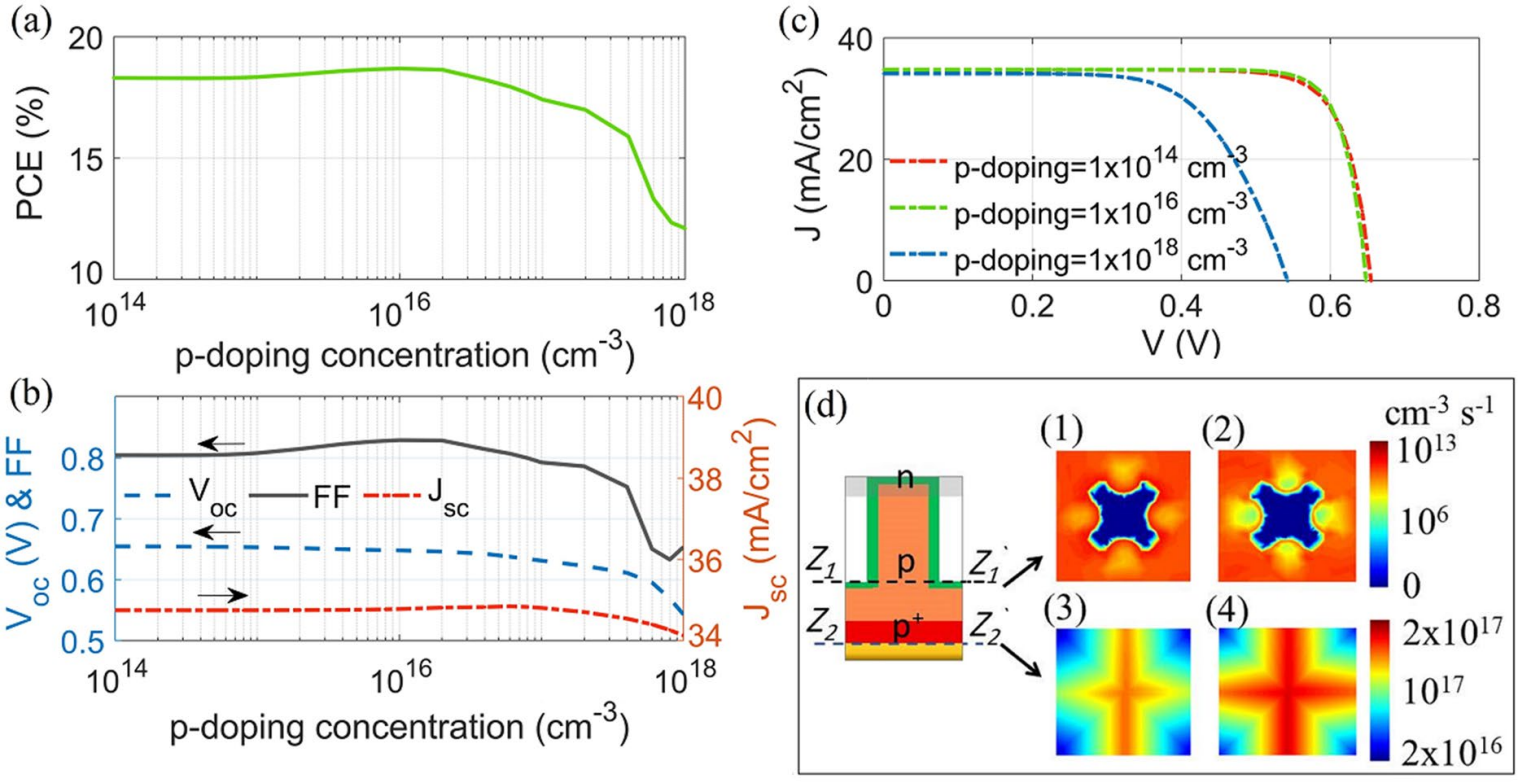
(**a**) PCE, (**b**) $V_{oc}$ , FF, and $J_{sc}$ versus the p-doped layer. (**c**) J–V characteristics, and (**d**) SR rate over NW sidewalls at NW-substrate interface (Z1–Z1′) at p-doping (1) $1\times 10^{16}$ $\hbox {cm}^{-3}$, and (2) $1\times 10^{18}$ $\hbox {cm}^{-3}$. The corresponding SR rate at BSF-metal interface (Z2–Z2′) is at (3) and (4). This image is created by Lumerical 2020a, FDTD Solver Version 8.23.2305, https://www.lumerical.com (license number-12802) released to Zewail City of Science and Technology, Giza, Egypt.

The p-doping layer is common with the top-NW (n/p) and the rear substrate ($p/p^+$) junctions. Therefore, the p-doping concentration has been varied separately to obtain the optimal performance of the two combined junctions. Figure 7a shows the graph of the PCE versus the p-doped layer level ($\hbox {N}_a$ = $1\times 10^{14} \text{cm}^{-3}$ to $1\times 10^{18}$ $\hbox {cm}^{-3}$), while the $V_{oc}$, FF, and electrical $J_{sc}$ are shown in Fig. 7b. As shown, the $V_{oc}$ and $J_{sc}$ are nearly constant over the range from $1\times 10^{14} \text{cm}^{-3}$ to $5 \times 10^{16}$ $\hbox {cm}^{-3}$, back to a long carrier diffusion length compared to the path length of the design proposed. It has also been shown that the maximum PCE of 18.7% can be obtained at a p-doping concentration of $1\times 10^{16}$ $\hbox {cm}^{-3}$ where the $V_{oc}$, $J_{sc}$ and FF are equal to 658 mV, 34.8 mA$/\hbox {cm}^2$ and 0.83, respectively. If $\hbox {N}_a$ is further increased to $1\times 10^{18}$ $\hbox {cm}^{-3}$, the values of $V_{oc}$ and FF were reduced to 550 mV and 0.63, respectively. Consequently, the value of PCE had dropped to 12%, with a reduction of 36%, which could be attributed to the reduced diffusion length with the doping level and hence the carrier lifetimes will drop. Therefore, the reverse bias leakage current is decreased and hence the values of $V_{oc}$ and FF^60^, where additionally, the PCE reduction is related mainly to the FF value, as shown in Fig. 7b. The FF reduction is 32%, while 18% and 2% are obtained for the $V_{oc}$ and the $J_{sc}$, respectively. This occured owing to the incorrect doping concentration which causes a poor field match between the two junctions, which is similar to introducing a resistance in the SCs^61,62^. This can produce a built-in voltage-drop at the maximum power point operation. Therefore, the peak power ($V_{p}$ $J_{p}$) is reduced, and hence the FF (see Eq. (3)).

Figure 7c shows the J–V characteristics at the p-doping core-substrate of $1\times 10^{16}$ $\hbox {cm}^{-3}$, and $1\times 10^{18}$ $\hbox {cm}^{-3}$. The corresponding doping effect on the SR rate is presented in Fig. 7d, where Fig. 7d1,d2 show the SR rate over the NW sidewalls, at the NW-substrate interface, with doping of $1\times 10^{16}$ $\hbox {cm}^{-3}$ and $1\times 10^{18}$ $\hbox {cm}^{-3}$, respectively. The depletion region shrinks with the NW doping concentration and therefore the SR rate over the sidewalls is reduced^49^. However, due to the small shell thickness of the design reported, the doping-based SR on the QCr-NW sidewalls can be seen to be insignificant (in the order of $\sim$ 10$^{13}$). On the other hand, Fig. 7d3,d4 present the effect of the p-doping layer over the rear metal contact, at doping levels of $1\times 10^{16}$ $\hbox {cm}^{-3}$, and $1\times 10^{18}$ $\hbox {cm}^{-3}$. At the optimum level of p-doping ($1\times 10^{16}$ $\hbox {cm}^{-3}$), the $p/p^+$ junction improves the reverse back voltage and thus this effectively reduces the SR rate, on the back metal (in the order of $10^{17}$), as shown in Fig. 7d3. Conversely, at the higher p-doping concentration ($1\times 10^{18}$ $\hbox {cm}^{-3}$), the reverse back voltage is minimized and thus the SR rate was increased on the back metal, as shown in Fig. 7d4. Moreover, the efficiency dramatically reduces at a doping level of $1\times 10^{18}$ $\hbox {cm}^{-3}$ which may also relate to the carrier lifetime reduction and hence the SRH recombination rate is increased^18^, especially in the bottom substrate junction. So tuning the p-doping layer is important, to obtain the optimum behaviour between the two junctions.

**Figure 8:**
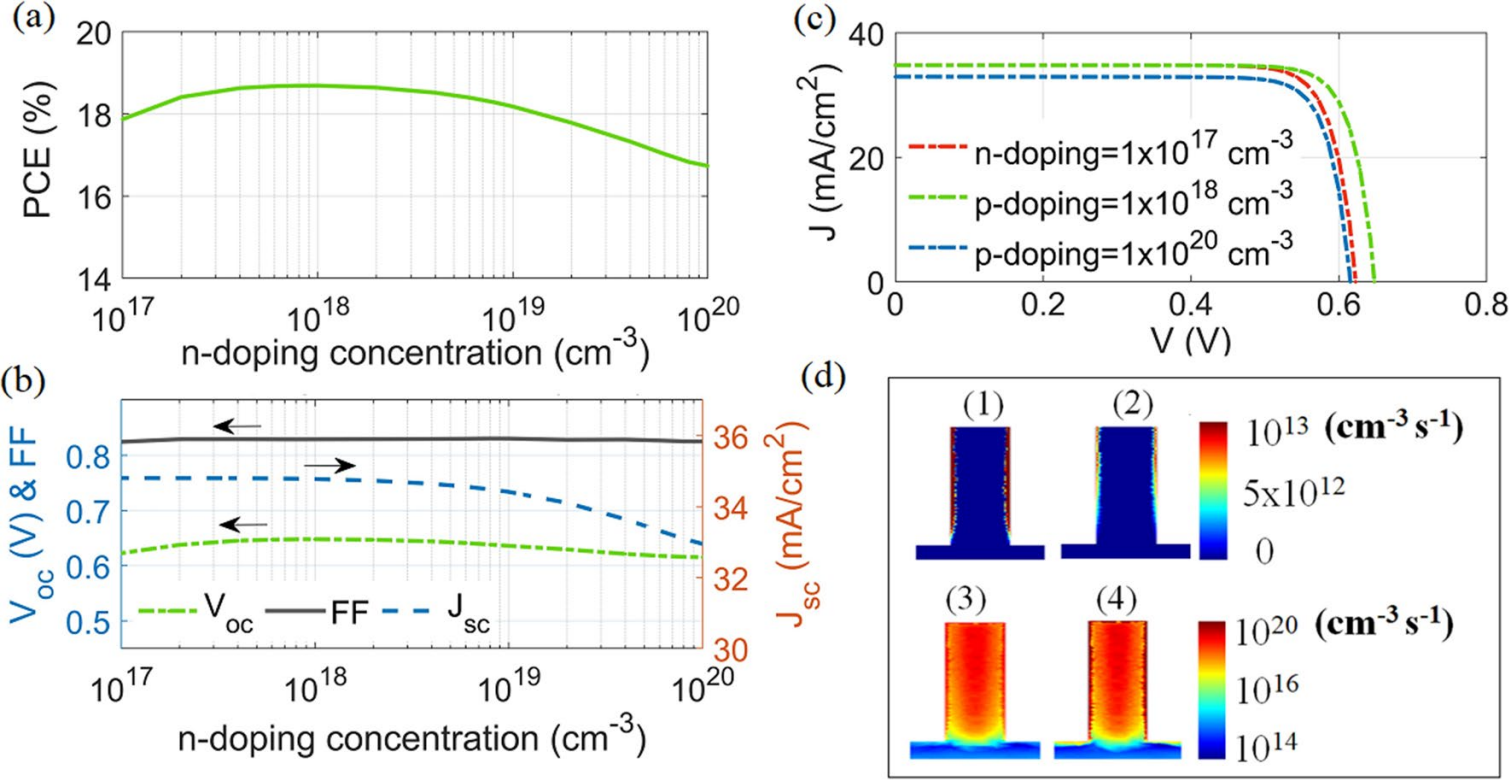
(**a**) PCE, (**b**) $\hbox {V}_{{oc}}$ , FF, and $\hbox {J}_{{sc}}$ versus the n-doped shell layer, (**c**) J–V characteristics and (**d**) SR rate for n-doping of (1) $1\times 10^{18}$ $\hbox {cm}^{-3}$, and (2) $1\times 10^{20}$ $\hbox {cm}^{-3}$, and the corresponding SRH rate is at (3) and (4) along the d–d′ plane (bottom substrate is not shown here). This image is created by Lumerical 2020a, FDTD Solver Version 8.23.2305, https://www.lumerical.com (license number-12802) released to Zewail City of Science and Technology, Giza, Egypt.

Figure 8a,b show the relationship between the PCE, and $\hbox {V}_{{oc}}$, FF and $\hbox {J}_{{sc}}$ as a function of the n-doping level of the shell layer (Nd = $1\times 10^{17}$ $\hbox {cm}^{-3}$ to $1\times 10^{20}$ $\hbox {cm}^{-3}$) respectively. It is evident that the PCE was not significantly changed by the doping changing from $1\times 10^{17}$ to $1\times 10^{19}$ $\hbox {cm}^{-3}$. This could be attributed to the smaller n-doped shell of the QCr-NW design proposed and thus its depletion region is small compared to the planar SC^18,48^. Therefore, the doping level has a weak effect on the junction performance^18^. It has been found that maximum PCE of 18.7% can be obtained at an optimum n-doped level (Nd), p-doped level ($\hbox {N}_a$), and $\hbox {p}^+$-doped BSF layer ($Na^+$) of $1\times 10^{18}$ $\hbox {cm}^{-3}$, $1\times 10^{16}$ $\hbox {cm}^{-3}$, and $4\times 10^{20}$ $\hbox {cm}^{-3}$ at SRV= $10^{20}$ cm/s, respectively. The corresponding values of $\hbox {V}_{{oc}}$, $\hbox {J}_{{sc}}$ and FF are 658 mV, 34.8 mA/cm${^2}$ and 0.83, respectively. If Nd is further increased to $1\times 10^{20}$ $\hbox {cm}^{-3}$, the value of $\hbox {J}_{{sc}}$ is reduced to 32.9 mA/cm${^2}$. This may relate to the reduced diffusion length and hence the bulk recombination is increased^18^. Figure 8c shows the J–V characteristics at n-doping shell values of $1\times 10^{17}$ $\hbox {cm}^{-3}$, and $1\times 10^{18}$ $\hbox {cm}^{-3}$, and $1\times 10^{20}$ $\hbox {cm}^{-3}$. Figures 8d1–d4 show the values of the SR and SRH recombination rates, respectively, along the d-d‘ plane at n-shell doping of $1\times 10^{18}$ $\hbox {cm}^{-3}$, and $1\times 10^{20}$ $\hbox {cm}^{-3}$. For a smaller doping level of $1\times 10^{18}$ $\hbox {cm}^{-3}$, the minority carriers were increased and therefore the SR rate along the NW sidewall was slightly increased to $1\times 10^{13}$ ${{\text{cm}}^{-3}\,{\text{s}}^{-1}}$, as shown in Fig. 8d1. However, the corresponding value of SRH recombination close to the NW sidewall is reduced, as shown in Fig. 8d3. For the high shell doping value ($1 \times 10^{20}$ $\hbox {cm}^{-3}$), the minority carrier decreases and therefore the SR rate is decreased. However, the SRH recombination rate was strongly increased to $1\times 10^{20}$ ${{\text{cm}}^{-3}\,{\text{s}}^{-1}}$, near to the NW surface. The optimum n-shell doping ($1\times 10^{18}$ $\hbox {cm}^{-3}$) relates to a lower total recombination rate and thus the conversion efficiency is maximized, as shown in Fig. 8a.

**Figure 9:**
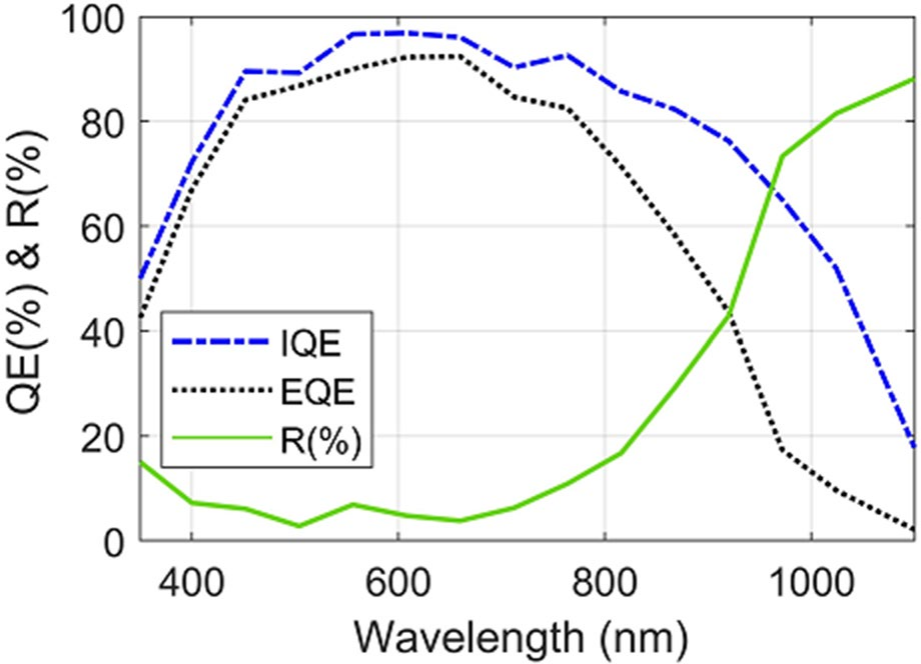
The Quantum efficiency (QE), and the Reflection loss in (%) versus the light wavelength.

To underline the implied mechanism of the power conversion enhancement, the quantum efficiency is investigated for the proposed design in the core–shell junction. Figure 9 shows the internal and external quantum efficiencies versus the light wavelength. To clarify the EQE behavior, the reflection spectra in percentage are included in the figure. For example, when all photons of a specific wavelength are absorbed and the generated electron–hole pair are collected, the quantum efficiency at this wavelength is unity. It is evident that the IQE of the shorter wavelengths and longer wavelengths is small compared to the visible wavelengths. The shorter wavelengths needs for a shorter path to get absorbed and therefore it can be found near to the NW surface. Therefore it get recombined near to the front surface along the NW structure or/and on the substrate surface between NWs. One the other hand, the longer wavelengths needs a longer path to be absorbed in the active material. Thus it suffers from the carrier recombination loss inside the active material (SRH recombination) in addition to the recombination on the rear surface. By applying the reflection loss (R) to the IQE, the EQE is calculated as shown in Fig. 9. We can see that the EQE is analog to the IQE behavior till wavelength of 750 nm and above this value the EQE is strongly dropped. To understand this, the reflection loss is involved in the Figure. It may be seen that the reflection is slightly changed in the range from 300 to 750 nm. However, reflection loss is strongly increased when the wavelength is further increased and therefore the EQE is strongly dropped above wavelength of 800 nm^63,64^.

### n-doped thickness effect

**Figure 10:**
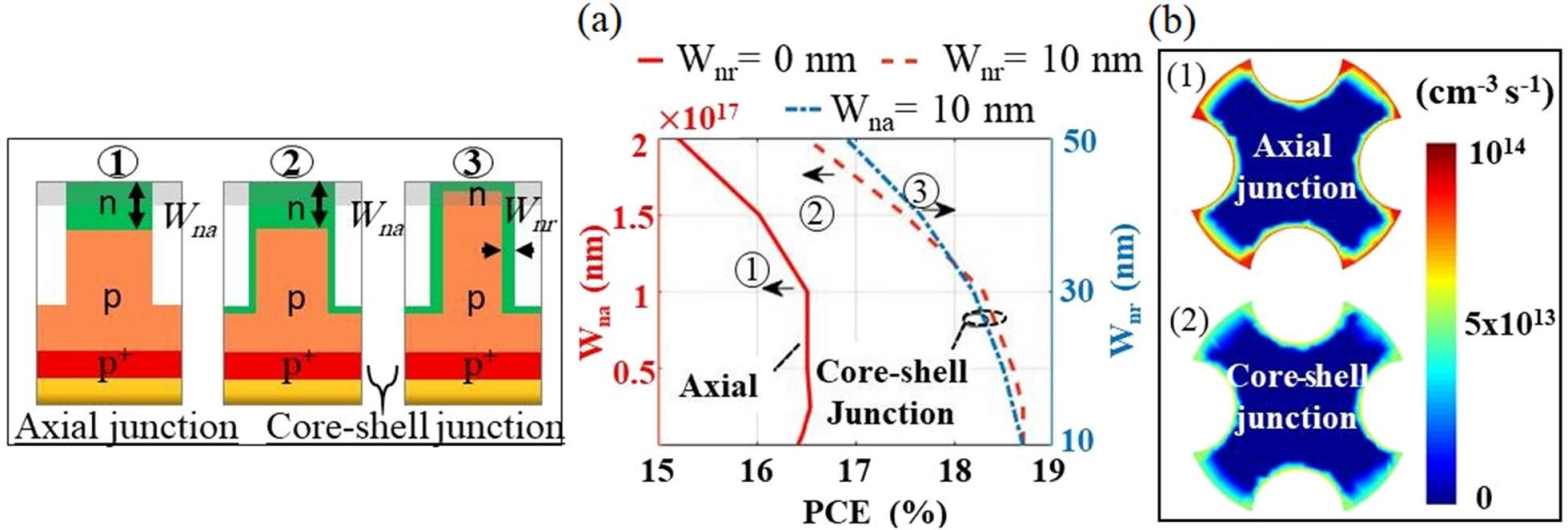
(**a**) PCE versus the thickness of n-top doped layers of the proposed design in the axial and core–shell junctions, (**b**) SR rate at $1\,\upmu \hbox {m}$ above the substrate for the proposed QCr-NW in (1) the axial and (2) core–shell junctions at the optimum doping concentrations. This image is created by Lumerical 2020a, FDTD Solver Version 8.23.2305, https://www.lumerical.com (license number-12802) released to Zewail City of Science and Technology, Giza, Egypt.

It is a considerable challenge to obtain a small radial shell thickness, without any overgrowth of the top axial shell layer^49^. Figure 10a shows the n-doped shell thickness as a function of the PCE value. Initially, the performance of design proposed (curve 1) has been investigated with no radial shell; Wnr = 0 (i.e. the axial junction). It can be observed that the value of PCE has dropped (solid red line) compared to the core–shell junction and has its maximum value of 16.5% at $\hbox {W}_{{na}}$ = 250 nm, to the standard thickness of the axial shell^65^. When $\hbox {W}_{{na}}$ is larger than 1000 nm, the n-doped layer becomes more effective and hence the lifetime was significantly reduced. Thus the recombination related to the SRH and the Auger mechanisms are increased^18^. If $\hbox {W}_{{na}}$ is further increased to 2000 nm, then the PCE value decreases to 15.2%.

Second, the performance of the core–shell junction has been studied by investigating the top-axial and radial shell layers separately. For the top-axial shell (curve 2), the PCE has been studied as a function of the top-axial shell width ($\hbox {W}_{{na}}$ = 10 nm to 2000 nm) at a fixed radial shell width (Wnr) of 10 nm. Figure 10a shows that for the top-axial shell of widths less than 250 nm, the PCE was nearly constant in value. Thus the core–shell junction of the design proposed was not strongly affected by considering the practical 110 nm-thick top-axial shell^49^. This may be attributed to the optimized n-doped layer that is not effusively doped ($1 \times 10^{18}$ $\hbox {cm}^{-3}$), and therefore the recombination was not further increased with the thickness. Moreover, there is a high lateral core–shell volume for the diffused carriers over the small radial path and hence an efficient extraction. Therefore, the induced radial electric field is dominant and so the photogenerated carriers are dominantly collected radially, offering a better performance. It can be deduced that the PCE for such a realistic study is 18.6%, with good surface passivation ($10^{2}$ cm/s). If $\hbox {W}_{{na}}$ is further increased to 2000 nm, the PCE decreases to 16.5%. This means the electric field force of the axial junction becomes comparable to the radial force (i.e. the generated carriers are strongly subjected to lower mobility and lower lifetime in the thicker n-doped axial shell). Therefore, the SRH and the Auger recombination effects increase, which decreases the value of the PCE. For the radial shell (curve 3), Fig. 10a shows also the variation of the PCE as a function of the change of the radial shell ($W_{nr}$ = 10 nm to 50 nm) at a fixed axial shell $\hbox {W}_{{na}}$ of 10 nm. It is evident that a small radial shell width is recommended, to reduce the recombination over the thick shell and obtain high levels of PCE of the core–shell junction^49^. The PCE decreases to 16.9% at a 50 nm thick radial width with a reduction of 9.5% compared to the 10-nm-thick n-doped radial shell. It may be concluded from this figure (Curves 2 and 3) that the proposed design offers a better performance, compared to the axial junction. This may be attributed to the small lateral path length of carriers, especially though the reported NW and hence the charge carriers can be extracted efficiently.

To show how the junction type can affect the NW surface performance of the design proposed, the SR rate profiles were investigated for both junctions. Figure 10b1,b2 show clearly the SR rate at 1 $\upmu$m above the substrate of the optimum proposed NW in the axial and core–shell junctions. The poor performance of the axial junction can be related to the minority carrier depletion over the NW radius and therefore the SR rate is increased, as shown in Fig. 10b1. Due to the n-doped shell layer, the minority carriers are diminished^57^ and besides, the radial junction confines the depletion of the minority carriers in a region near the NW surface^57^. Therefore, the SR rate is reduced at the surface, compared to the axial junction.

Interfacial defects contain external surfaces, stacking faults, and grain boundaries. the stacking faults and grain boundaries are two foremost types of interfacial defects. The grain boundaries are present in polycrystalline materials. Grain boundaries results from different atoms grow simultaneously in specific positions with different orientations as a consequence of metal solidification. It can be considered as one of surface defects as they separate atoms with different crystallographic orientations^66^. Moreover, impurities (either intentional or unintentional) in the active material produce trap states. Moreover, the most active material have energy levels close the middle of the band gap. In addition, defects in the active material form unoccupied “trap” states within the bandgap. Recombination process occurs as the electron relaxes to the trap state from the conduction band where its energy is transferred to the lattice or in a photon emission. In the same way, the hole from the valence band moves to the same trap state^67,68^. We can say that these defects affect the lifetime of the carriers generated, recombination rate and hence the PCE. In this regard, the bulk trap-assisted (SRH), the radiative (Rad) and Auger (Au) based recombination are employed in this study. Furthermore, the effect of recombination (SR) along the NW surface and on the metal interface is included^67,68^.

### Radiative recombination effect

**Figure 11:**
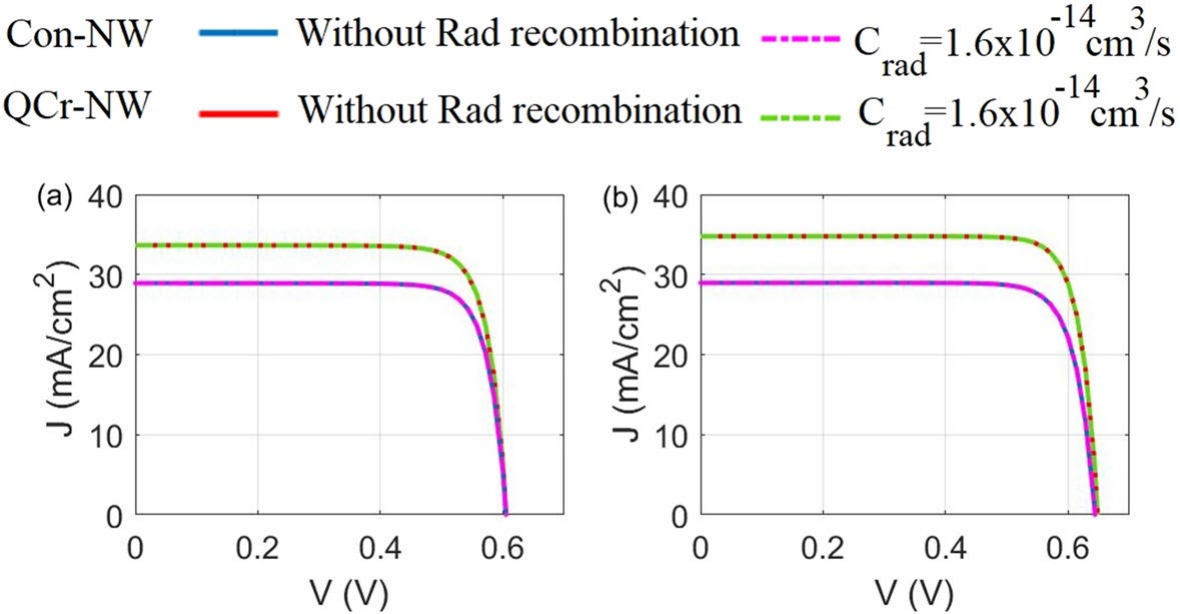
J–V characteristics with and without radiative recombination for the studied designs in the (**a**) axial and (**b**) core–shell junctions.

Radiative recombination transitions are usually significant only in materials of a narrower bandgap, or/and a bandstructures which permit direct transitions in momentum^69^. Most of SCs are made from silicon semiconductor (an indirect bandgap) and therefore radiative recombination is very low and frequently neglected^69^. To get clear of this in the studied NWs, Fig. 11a,b show the J–V characteristics for the proposed and conventional NW designs in the axial and core–shell junction with and without including the radiative recombination. It is evident that the electrical behavior for the two studied designs is identical whether for the axial or the core–shell junction. This is back to the small coefficient of the radiative coefficient besides to the small geometry of the proposed designs. The PCE in the axial junction with or without the radiative recombination is equal to 18.7% and 15.1% for the proposed and the conventional NW designs, respectively. However, 18.7% and 15.1% for the proposed and the conventional NW designs in the core–shell junction.

### Trap-assisted (SRH) effect

**Figure 12:**
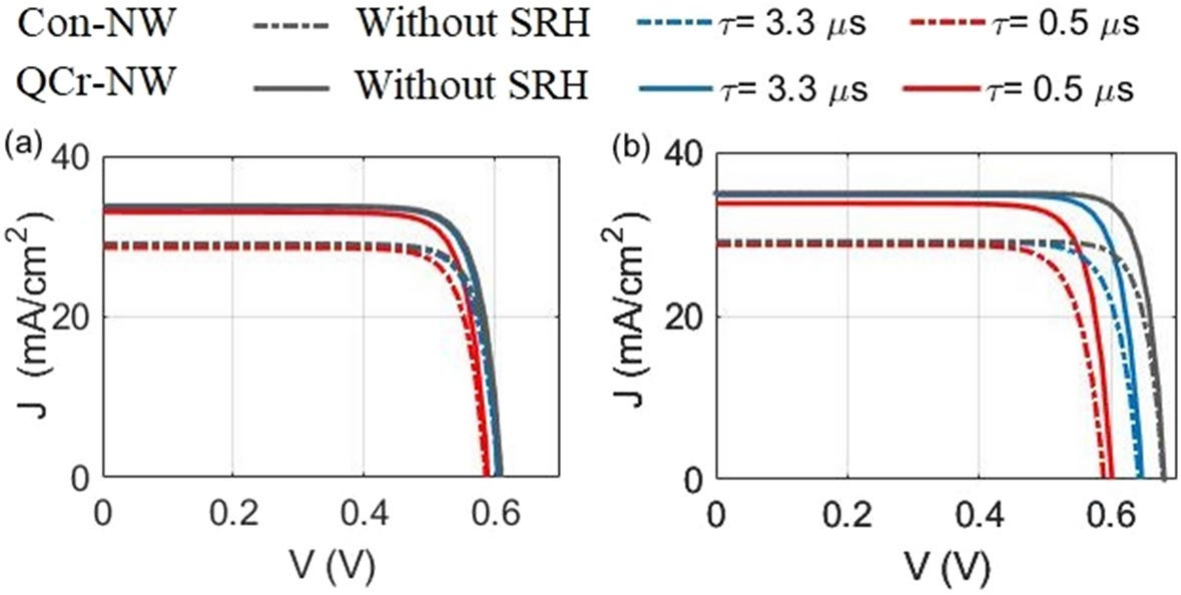
J–V electrical characteristics for both the studied designs in (**a**) the axial and (**b**) core–shell junctions at different lifetime values.

The charge carrier lifetime is one of the most significant parameters in solar cells. It shares with the carrier mobility a key role in defining the carrier diffusion length and hence in identifying the optimal thickness of the device^32^. In the previous sections, the carrier lifetimes for electron and holes have been seen to change with the doping level, via the built-in Fossum model^70^. To visualize how the SRH carrier lifetime influences the junction performance, the (J–V) electrical characteristics have been investigated with and without the trap-assisted recombination effect. Figure 12a,b show the PCE of the studied designs in the axial and core–shell junctions, respectively, at different lifetime’s values. In this analysis, the doping concentrations of the layers are fixed at the optimum values. Of the Figs. shown we note that the $J_{sc}$ of the studied designs in both junctions is slightly reduced with the carrier lifetimes. It can be attributed to the carrier path length is comparable to the diffusion path length of the carrier^32^. This may back to the small geometry of the active material, besides the light doping of the large core region ($1\times 10^{15}$) $\hbox {cm}^{-3}$ as reported in Ref.^71^. Moreover, the highly doped thickness of the studied designs thickness are small and therefore the diffusion length exceed the active material thickness^71^. For the axial junction (Fig. 12a), it is seen that the reduction in the J–V curve is nearly constant for both the studied designs. On the other hand, the reduction in the $J_{sc}$ of the proposed QCr-NW is slightly increased compared to the Con-NW in the core–shell junction (Fig. 12b). This may back to the increased n-doping radial shell volume of the offered design compared to the conventional cylindrical shell in the core–shell junction. Moreover, the value of $V_{oc}$ of the core–shell junction of both studied designs was noticeably decreased compared to the axial junction with the reduction in the value of the lifetime. This may back to the decreasing the surface recombination rate, and hence the effect of the bulk recombination is increased compared to in the axial junction^72^. In addition, the holes minority carriers’ life time is reduced due to the highly n-doped shell layer. Therefore, the higher saturation current density-dependent recombination is increased in core–shell junction^49^.

The corresponding maximum value of the PCE of the axial and core–shell junctions of the studied designs is shown in Table 4. Without taking the SRH recombination into consideration, the PCE for the proposed QCr-NW and Con-NW structures in the axial junction is increased to 16.8% and 14.5%, respectively. However, 20% and 16.6% is obtained for core–shell junction of the studied designs. As the lifetime ($\tau _o$) decreases in the conventional design to 3.3 $\upmu$s and 0.5 $\upmu$s, the PCE reduces to 14.2% and 13.6% in the axial and to 15.1% and 13.4% in the core–shell junctions, respectively. However, to 16.5% and 15.8%, and to 18.6% and 16.5% are obtained for the proposed design in the axial and core–shell junction, respectively. Thus, the core–shell junction still offers a higher PCE value compared to the axial junction at the lower lifetime value. Moreover, the proposed crescent NW provides a PCE higher than the conventional design in both axial and core–shell junctions. It is worth noting that the carrier lifetime change has a weak effect on the QCr-NW design proposed, compared to the doping concentration of the junction^49^ shown in Figs. 5, 7 and 8.

**Table 4:** The PCE of the proposed and conventional NW designs in the axial and core–shell junction at different lifetime values.

SRH lifetime ($\upmu$s)	Axial junction			Core–shell junction		
	$\tau _o$ = 0.5 $\upmu$s	$\tau _o$ = 3.3 $\upmu$s	Without SRH effect	$\tau _o$ = $0.5\, \upmu \hbox {s}$	$\tau _o$ = 3.3 $\upmu$s	Without SRH effect
$PCE\, (\%)_{QCr-NW}$	15.8	16.5	16.8	16.5	18.5	20
$PCE \,(\%)_{Con-NW}$	13.6	14.2	14.5	13.4	15.1	16.6

### Auger recombination effect

**Figure 13:**
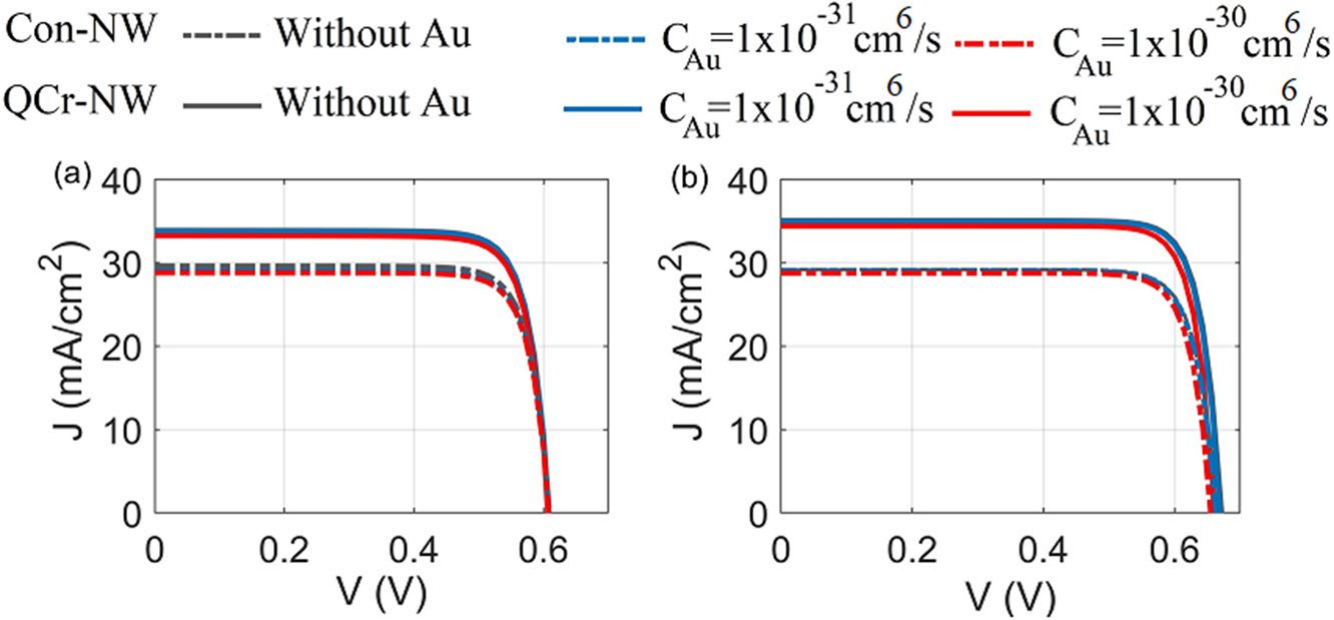
J–V characteristics under different Auger coefficient for the studied designs in the (**a**) axial and (**b**) core–shell junctions.

Auger recombination (AR) plays a main process for TF Si SCs in high quality c-Si when the doping exceeds $1\times 10^{17}$ $\hbox {cm}^{-3}$ as reported in^71^. It depends only on the carrier density or heavy doping different from other recombination processes. In this mechanism, electrons and holes recombine in a band-to-band transition; however the resulting energies are given off to other electrons in the conduction band or holes in the valence band respectively^67,68^. Sinton and Swanson^73,74^ accurately measured the Auger coefficients for hole ($C_{p}$) and electron ($C_{n}$) minority carriers at an excess carrier density range between $1\times 10^{15}$ $\hbox {cm}^{-3}$ and $2\times 10^{17}$ $\hbox {cm}^{-3}$. It is found to equal four times ($1.66\times 10^{-30}$ $\hbox {cm}^{6}\,{\text{/s}}$^73,74^ higher than the more cited values of Dziewior and Schmid^75^. This conventional Auger theories are in good agreement with the lifetimes of highly doped silicon measured^73^. In this study, the PCE is investigated for the studied designs with and without taking Auger recombination into account. The electron and holes minority carriers lifetime of SRH is fixed at nearly 3.3 $\upmu$s and 4 $\upmu$s^55^, respectively, and under a good practical surface passivation of $10^{2}$ cm/s^27,33^. In this study, the Basbore model^72^ based the CHARGE solver^68^ is chosen to simulate the Auger recombination in the proposed and conventional designs. Figure 13a,b show the J–V characteristics of the proposed design compared to the conventional NW in the axial and core–shell junctions, respectively, with and without Auger recombination mechanism. The corresponding PCE of the proposed and conventional NW designs is shown in Table 5. Without the Au recombination, the PCE of the proposed crescent is increased to 16.7% and 19.6% for the axial and core–shell junction, respectively. However, 14.7 and 16% are obtained for the conventional NW in the axial and core–shell, respectively. It may be seen that the PCE of the studied designs in both junctions are decreased with the Auger recombination. The PCE of the proposed and conventional NW design is decreased to 16.3% and 14.2% in the axial junction, respectively, at the Auger Coefficient of $1\times 10^{-30}$ $\hbox {cm}^{6}\,{\text{/s}}$. However, 18.8% and 15.4% for the proposed and conventional NW design in the core shell junction. It is evident that the quad crescent structure in core–shell junction (Fig. 13b) suffers the high power reduction of 4% with increasing the Auger coefficient to $1\times 10^{-30}$ $\hbox {cm}^{6}\,{\text{/s}}$. This may back to the increased n-doping shell volume for the proposed crescent NW design compared to in the conventional NW design at the core–shell junction. The Auger lifetime is decreased with the doping concentration and hence the Auger recombination rate is increased^71^. On the other hand, the proposed structure in the axial junction ( Fig. 13a) offers the lower power reduction of 2% due to the smaller n-doping top volume. Consequently, both the trap-assisted based SRH and the Auger recombination mechanisms limit the lifetime and diffusion length of Silicon NWSCs^67^.

**Table 5:** The PCE of the proposed and conventional NW designs in the axial and core–shell junction with and without AR mechanism.

Au coefficient	Axial junction			Core–shell junction		
	Without Au	$1 \times 10^{-31}$ $\hbox {cm}^{6}/s$	$1\times 10^{-30}$ $\hbox {cm}^{6}/s$	Without Au	$1\times 10^{-31}$ $\hbox {cm}^{6}/s$	$1\times 10^{-30}$ $\hbox {cm}^{6}/s$
$PCE \,(\%)_{QCr-NW}$	16.8	16.7	16.4	19.62	19.5	18.95
$PCE \,(\%)_{Con-NW}$	14.7	14.4	14.3	15.94	15.8	15.5

### Surface recombination (SR) effect

**Figure 14:**
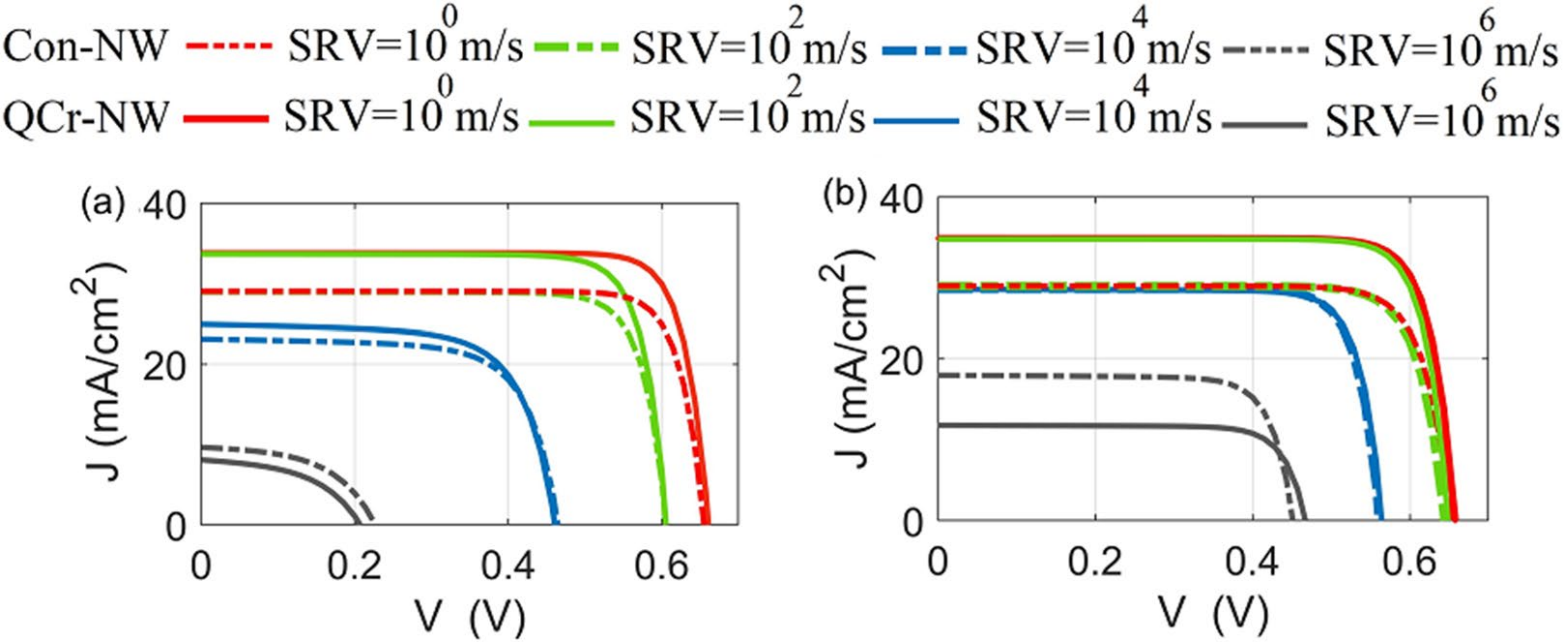
J–V electrical characteristics for both the studied designs at each of (**a**) the axial and (**b**) core–shell junctions at different SRVs values.

Figure 14a,b show the J–V characteristics for the axial and core–shell junctions, respectively, at different SRVs. Firstly, it can be deduced from this figure that the PCE of the core–shell junction is high, compared to the axial junction all over the SRV values studied. The core–shell junction enhancement may be related to the small aspect ratio of the radial path in the core–shell design, compared to the path in the axial junction. Therefore the photo-generated carriers are diffused, with a small lateral length and thereby collected efficiently^32,49^. In the case of ideal passivation (SRV $\sim$ 1 cm/s), the PCE of the proposed QCr-NW design for the axial and core–shell junctions are given by 18.5% and 19%, respectively, while these value are 15% and 15.4% for the solid NW studied. The QCr-NW design proposed offers an enhancement of 23% for both the axial and core–shell junctions. It is worth noting that the PCE of the axial junction lies above the efficiency of the asymmetric NW with a crescent nanohole in the work of Khaled et al.^34^. This offers an enhancement of 8%, with a volume reduction of 45%, compared to the crescent nanohole design reported. Table 6 shows the PCE for the proposed QCr-NW and conventional designs for both junctions, at SRV $\sim$ 1 cm/s.

The SR rate plays a significant role in the performance of the NWs TF SCs studied^49^. In this work, the surface roughness is modelled by defining a surface recombination velocity at the SiO2 interface of the proposed design^76^. The results from Fig. 14 show that junctions for both the studied designs experience a reduction in the values of $\hbox {J}_{{sc}}$ and $\hbox {V}_{{oc}}$ with SRV. There is always a competition between improved of optical absorption and increased of surface recombination^77^. In this study for a practical behavior, a SRV value of $10^{2}$ cm/s was taken as suggested in Ref.^27^. The maximum PCE was decreased to 16.5% and 18.7% for the design proposed, with reductions of 1.6% and 11% in the axial and core–shell junctions, respectively. On the other hand, the PCE of the conventional NW in core–shell and axial are decreased to 15.1% and 14.2% with a reduction of 1.3% and 10%, respectively. Consequently, the enhanced absorption can dominate over surface recombination compared to the conventional NW structure. Conversely, the effect of the surface recombination is strongly increased at the bad passivation and the decrease of both $\hbox {V}_{{oc}}$ and $\hbox {J}_{{sc}}$ becomes significant for both junctions, as shown in Fig. 14. However, the PCE of the axial junction was strongly influenced when compared to the core–shell junction. For the axial junction, the PCE of the suggested design was significantly decreased to 7.9%, and 0.8% for SRVs of $10^{4}$ cm/s and $10^{6}$ cm/s, respectively. This is due to the rapid drop of all the electrical parameters. The values of the $V_{oc}$ , FF, and $J_{sc}$ were decreased by 68%, 42%, and 76% at values of the SRV of $\sim$ 10$^6$ cm/s. Therefore, the corresponding maximum PCE was reduced to 0.8% with a reduction of 96%. This is due to the electron quasi-Fermi level in the axial junction that is uniformly shifted to the valence band over the entire NW radius. Therefore, the axial junction was strongly influenced by the SR^57^. On the other hand, the conventional design is slightly affected compared to the high surface-related proposed design under the bad surface passivation values. At the SRV of $10^{4}$ cm/s, the PCE of the conventional design in the axial junction is equal to 7.5% close to the proposed design. When the SRV is further increased to $10^{6}$ cm/s, the PCE of the Con-NW is equal to 1.1% exceeding the proposed design by 27%. This may be related to increasing of the lateral surface area of the proposed design compared to the conventional NW design.

**Table 6:** The maximum electrical characteristics ($V_{oc}$, $J_{sc}$, FF and PCE) of the axial and core–shell junctions for the proposed QCr-NW and conventional solid NW structures at SRV $\sim$ 1 cm/s.

Structure	Axial junction				Core–shell junction			
	$\hbox {V}_{{oc}}$ (mV)	$\hbox {J}_{{sc}}$ (mA$/\hbox {cm}^2$)	FF (%)	PCE (%)	$\hbox {V}_{{oc}}$ (mV)	$\hbox {J}_{{sc}}$ (mA$/\hbox {cm}^2$)	FF (%)	PCE (%)
QCr-NW	660	83.8	83	18.5	660	34.9	83	19
Solid NW	650	29.0	81	15	65.3	29.0	82	15.4

For the core–shell junction, only in the region closer to the NW surface (the n-doped shell), is the hole quasi-Fermi level shifted to the conduction band. Thus, the hole minority carriers in the n-shell layer were trapped in a small region towards the NW surface^57^. Therefore, the core–shell junction reduces the impact of the SR on the electrical performance, especially for the values of $\hbox {V}_{{oc}}$ and FF, as shown in Fig. 10b. The PCE values of the proposed design decrease to 13.1%, and 4.3% for SRVs of $10^{4}$ cm/s, and $10^{6}$ cm/s, respectively. At a SRV of $10^{6}$ cm/s, the reductions of the $\hbox {V}_{{oc}}$, FF, and the electrical $\hbox {J}_{{sc}}$ are 29%, 6%, and 66%, respectively. Therefore, the maximum PCE of core–shell reduces by 77%, which verifies that the proposed design is preferable significantly, in the core–shell junction compared to the axial junction. The low $\hbox {V}_{{oc}}$ resulted from interfacial recombination is due to the large dark current^78^. Moreover, the large surface to volume ratio of the proposed NW design is expected to worsen recombination, making surface passivation more important for these geometries than for planar silicon solar cells^78^. On the other side, the conventional design is small influenced by the bad surface passivation compared to the proposed design. At SRV of $10^{6}$ cm/s, the PCE of the conventional design in the axial junction is equal to 13.1% identical to the proposed design. For a bad passivation above this value, the conventional NW compensates the enhancement of the optical power absorbed by the proposed design. At the SRV of $10^{6}$ cm/s, the PCE of the conventional design equal to 6.3% surpass the proposed design by 46%. Consequently, it is critical to obtain a good surface passivation especially for designs which have high surface area. Table 7 shows the PCE for the proposed QCr-NW and conventional designs for both junctions, at different SRVs values.

**Table 7:** The PCE of the proposed and conventional NW designs in the axial and core–shell junction at SRVs values.

Structure	Axial junction				Core–shell junction			
	SRV	SRV	SRV	SRV	SRV	SRV	SRV	SRV
	$10^{0}$ m/s	$10^{2}$ m/s	$10^{4}$ m/s	$10^{6}$ m/s	$10^{0}$ m/s	$10^{2}$ m/s	$10^{4}$ m/s	$10^{6}$ m/s
$PCE (\%)_{QCr-NW}$	18.5	16.5	7.9	0.8	19	18.7	13.1	4.3
$PCE (\%)_{Con-NW}$	15.8	14.2	7.5	1.1	15.3	15.1	13.1	6.3

**Figure 15:**
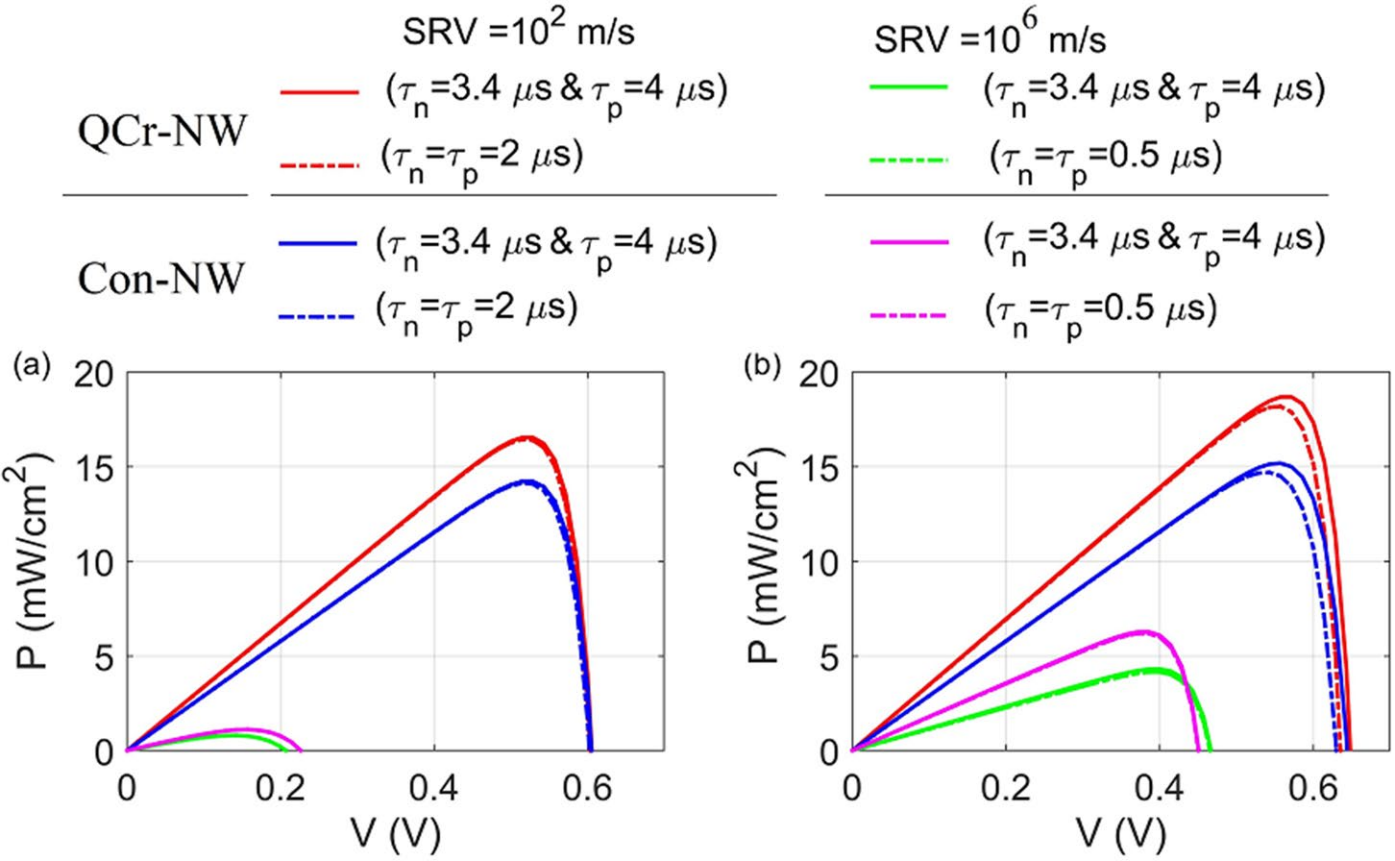
P–V characteristics for the studied designs at good ($10^{2}$ m/s) and bad ($10^{6}$ m/s) surface passivation with and without lifetime-dependent surface defects for the (**a**) axial junction and (**b**) core–shell junction.

In the previous study, the J–V characteristics were performed under different passivation value without taking into account the lifetime degradation. On the other hand, the processes of the MACE leads to dislocations and dangling bonds resulting in a severe SRH recombination^79^. It was reported that the carrier lifetime strongly depends on the injection level and the MACE etching time^79^. Further, the carrier lifetime decreases by increasing the frontal surface area in practical^79^. To investigate how the surface defects affects the reported NWs structures, the lifetime degradation (LTD) is taken into account. Figure 15a,b show the P–V performance for the reported designs under surface passivation with and without the lifetime-depending surface effects for the axial and core–shell junctions, respectively. In this study, the minority carrier lifetime of electron and holes are taken by 3.3 $\upmu$s and 4 $\upmu$s without taking the lifetime-dependent surface defects into account^76^. After considering the LTD, the carrier lifetime is taken by 2 $\upmu$s and 0.5 $\upmu$s at good and bad passivisation, respectively. In general, the lifetime-depending surface defects have a small effect on the electrical performance compared to the SR effect for the studied designs in both junctions. Table 8 shows the corresponding PCE of the conventional and proposed crescent structure with and without LTD under good and bad passivation. It is evident that the reduction in $P_{m}$ is noticeably increased at good passivation compared to the bad passivation^72^. This can be clearer at the core–shell junction as shown in Fig. 15b. For the axial junction, the proposed Cr-NW and Con-NW are slightly reduced to 16.4% and 14.1%, respectively, at the good passivation with the LTD. However, 0.8% and 1.1% are obtained at the bad surface passivation. On the other hand, the reported Cr-NW and Con-designs are decreased to 18.1% and 15.2% at good passivation and the LTD consideration, respectively, for the core–shell junction. However, 4.1% and 6.2% are obtained at the bad surface passivation. It is concluded that the $P_{m}$ is strongly influenced by the surface recombination compared to the surface effects LTD highlighting the significance of proper surface passivation of NWSCs^49^.

**Table 8:** The PCE of the conventional and proposed crescent structure in the axial and core–shell junctions with and without lifetime degradation under good and bad passivation.

	SRV	Axial junction		Core–shell junction	
		Without LTD	With LTD	Without LTD	With LTD
$PCE \,(\%)_{QCr-NW}$	$10^{2}$ cm/s	16.5	16.4	18.7	18.1
$PCE \,(\%)_{QCr-NW}$	$10^{6}$ cm/s	0.8	0.8	4.3	4.1
$PCE\, (\%)_{Con-NW}$	$10^{2}$ cm/s	14.2	14.1	15.2	14.4
$PCE\, (\%)_{Con-NW}$	$10^{6}$ cm/s	1.1	1.1	6.3	6.2

**Figure 16:**
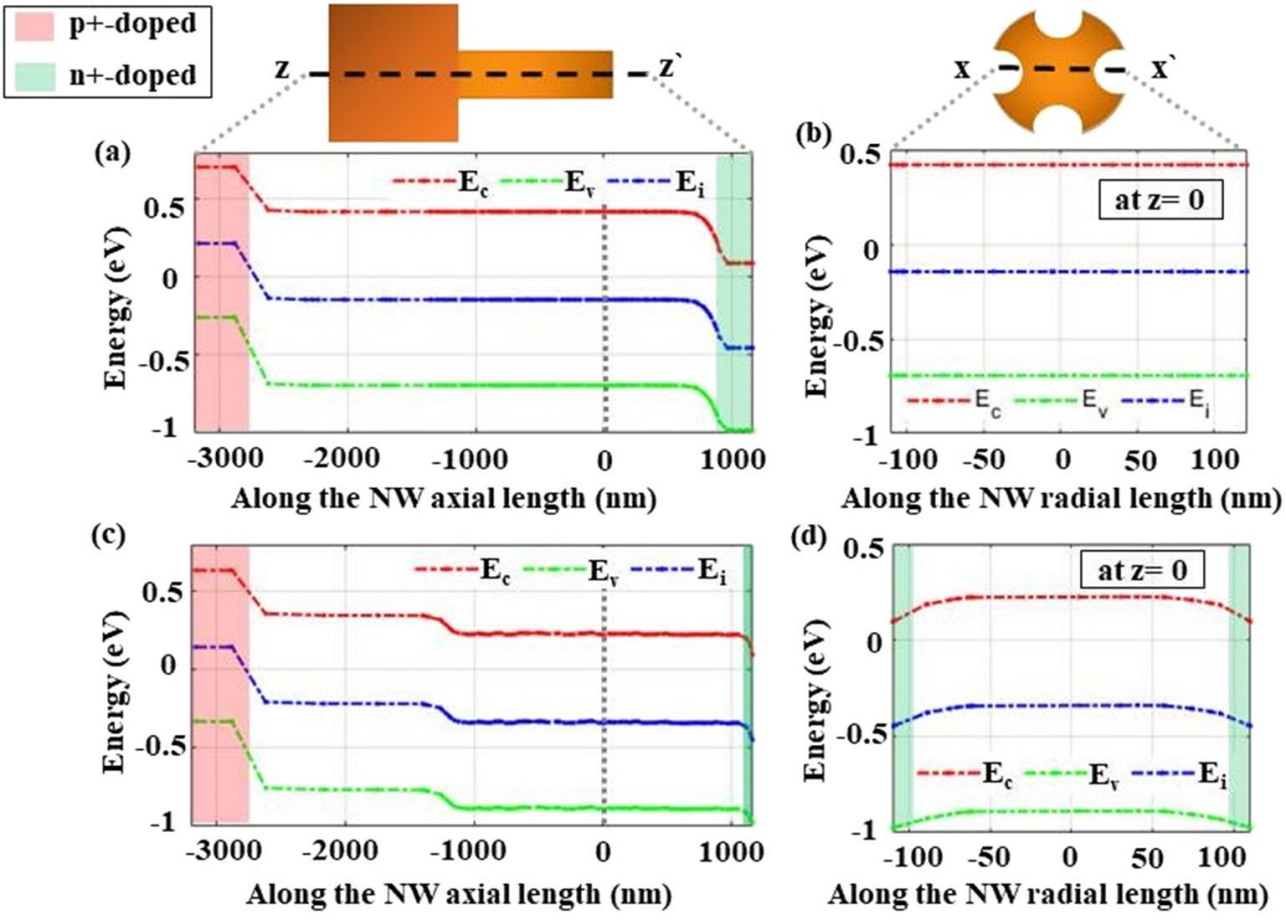
Band diagram along the (**a**)/(**c**) radial axis and (**b**)/(**d**) axial axis of the proposed NW in the axial and core–shell junction at the maximum power point operation. The water color of green is for the n-doped layer and the red is for the highly p-doped BSF layer.

Figure 16a,b show the radial and axial band diagram of the axial junction and Fig. 16c,d for the core–shell junction, respectively. The red, blue and green colors are for the conduction ($\hbox {E}_{{c}}$), intrinsic ($\hbox {E}_{{i}}$), and valence band ($\hbox {E}_{{v}}$), respectively. It is evident that in the core–shell junction, the depletion region is improved along the axial (zz’) direction, near the NW-substrate interface, due to n-doping of the substrate top. So, this thin shell introduces a better field match (i.e. reduces the SC internal resistance^61,62^) between the top (*n*/*p*) and rear ($p/p^+$) junctions. Consequently, it improves drifting in account of diffusion of carriers generated at the middle of the NW (z $\sim$ 1000 nm) compared to in the axial junction shown in Fig. 16a. Moreover, it can be seen that the radial shell introduces a voltage difference along the proposed NW length as shown in Fig. 16d. This helps in improving the collection probability of the electron carriers in radial compared to in the axial junction (Fig. 14b). Additionally, the high n-doped shell supports in reducing the effect of the recombination of holes minority along the NW surface. As a result, the $\hbox {J}_{{sc}}$ and hence the $\hbox {V}_{{oc}}$ is increased for the core–shell junction compared to the axial junction as shown at the good passivation shown in Fig. 14.

**Figure 17:**
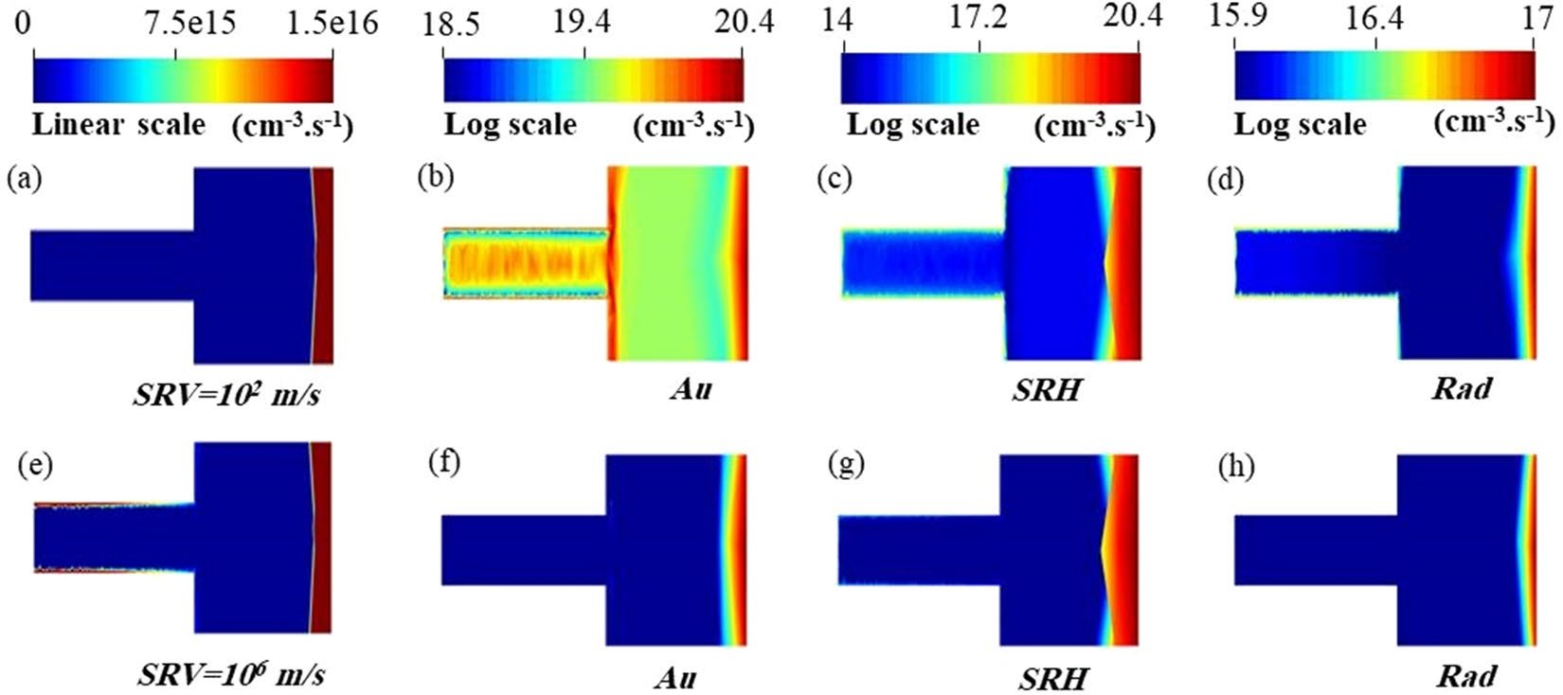
Recombination rate of the (**a**)/(**e**) surface, the (**b**)/(**f**) Au, the (**c**)/(**g**) SRH, and the (**d**)/(**h**) radiative for the proposed NW design in core–shell junction at good ($10^{2}$ m/s)/bad ($10^{6}$ m/s) surface passivation. This image is created by Lumerical 2020a, FDTD Solver Version 8.23.2305, https://www.lumerical.com (license number-12802) released to Zewail City of Science and Technology, Giza, Egypt.

  The SC band diagram strongly affect the recombination rate and hence solar cell electrical performance^80^. Figure 17a–h show the different recombination rate inside the proposed design in core–shell junction at the good surface passivation of $10^{2}$ m/s and bad surface passivation of ($10^{6}$ m/s), respectively. It can be deduced that, the recombination rate inside the active material is badly affected by the doping concentration especially for the auger and the SRH mechanisms in the core-shell junction compared to the axial junction. Moreover, the surface recombination affects all other recombination rate mechanisms^72^. At the good surface passivation, the probability of carriers to recombine inside the active material is increased as shown in Fig. 17b–d especially for the Au mechanism. However, the surface recombination rate is strongly increased by order of three along the NW without surface passivation as shown in Fig. 17e. The PCE is increased to 18.7% with the good surface passivation compared to 4.4% without the surface passivation. Consequently, a good surface passivation is important to convert the enhancement of the optical light power absorbed into electrical output power. Therefore, both the trap-assisted based SRH and the Auger recombination mechanisms influence carrier lifetime and recombination compared to the radiative mechanism in the NWSCs^67,72^.

**Figure 18:**
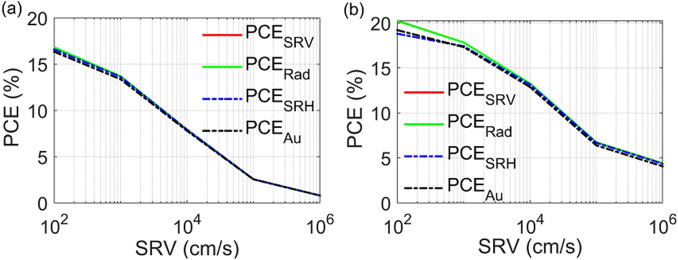
The PCE of the proposed and conventional NW designs in the axial and core–shell junction for Rad, SRH, and Au recombination mechanisms separately under different SRVs values.

It is worth noting that the SRV affects the recombination rate of other mechanisms^72^. To examine further how the SRV influence the rate of recombination of other mechanisms, the PCE is studied with including the trap-as sited (SRH), the Auger (Au), and the radiative (Rad) recombination separately. Figure 18 shows the J–V characteristics under the SRH, the Au, and the Rad recombination separately under different SRVs values. In general, the SR (red color) strongly affects the performance of the proposed NW structure compared to other recombination mechanisms. Additionally, the reduction in the PCE due to the trap assisted SRH and Au mechanisms is higher at the good passivation compared to in the bad surface passivation^72^. However, the contribution of the radiative recombination can be neglected compared to the other mechanisms^72^ as shown in Fig. 18.

Wang et al.^81^ have found that Young’s modulus (GPa) decreases with the temperature (K) increase for Si nanowires. The environmental conditions like wind, pressure and temperature affect the shear modulus and hence the stress on the solar cell in turn^81,82^. The proposed quad-crescent design has no variation in z-direction and can be fabricated through a single-step etching process same as the conventional NW structure using the Metal assisted chemical etching (MACE) technique^35^. However, the small geometry of the proposed NW may cause an increased pressure on it due to the top ITO layer. This problem can be solved by a thermal annealing crystallization step^78^ and filling the structure spaces by the $SiO_{2}$ material that works also as a surface passivation layer.

## Fabrication method

**Figure 19:**
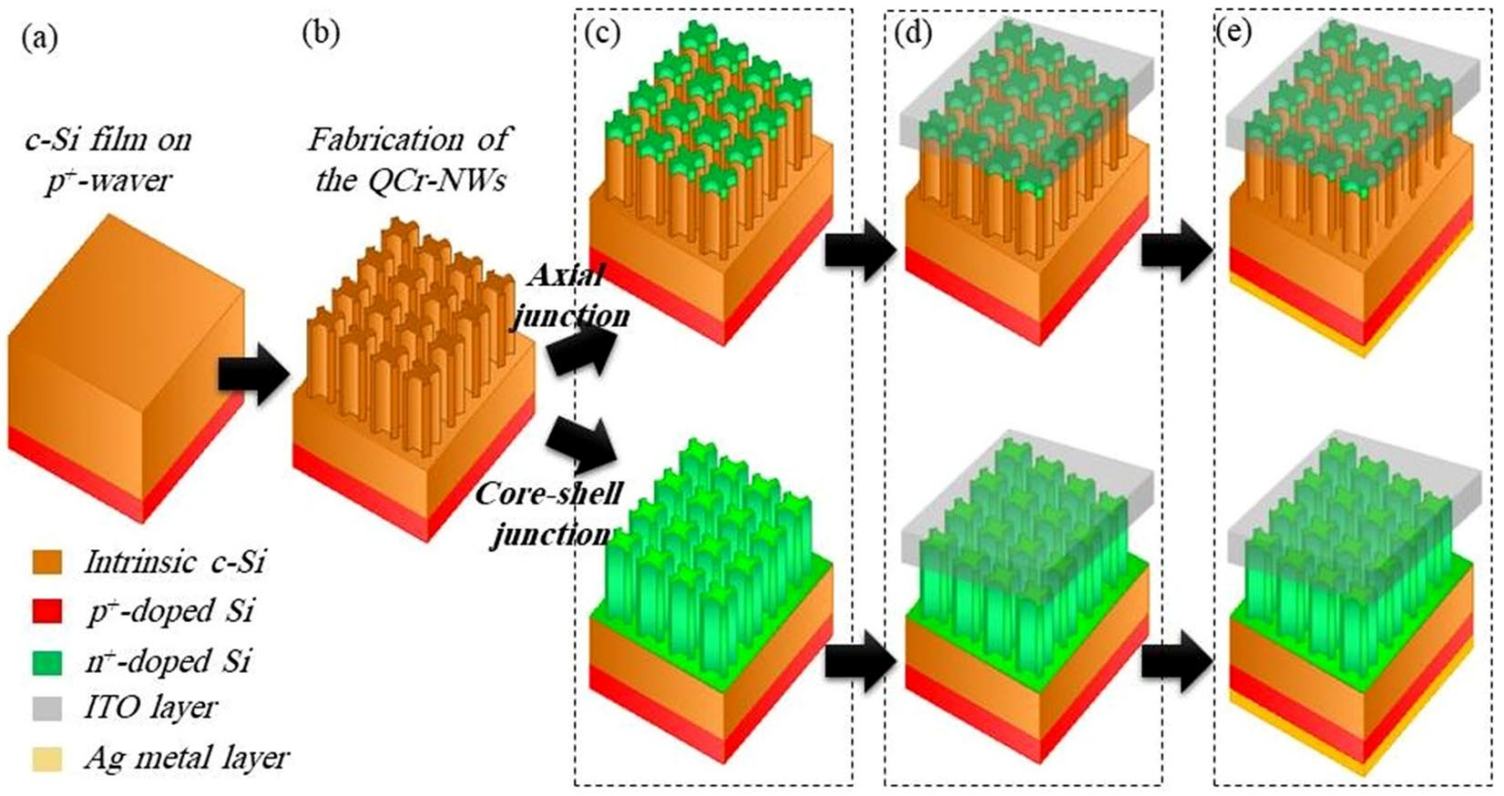
Fabrication steps proposed for the informed QCr-NW structure. This image is created by Lumerical 2020a, FDTD Solver Version 8.23.2305, https://www.lumerical.com (license number-12802) released to Zewail City of Science and Technology, Giza, Egypt.

The doping profiles of the design reported can be obtained by using the following steps. First of all, the optimal $\hbox {p}^+$-doped boron (B) atoms are diffused into the backside of the wafer to form the back surface field (BSF) layer^29^. Moreover, Sahoo and Kale had proposed that the c-Si layer can be grown epitaxially on top of the heavily doped Si substrate using a thermal chemical vapor deposition (RTCVD) reactor^21^. Different methods, such as electron beam, reactive ion etching or holographic lithography can be used for precise control of the shape, size and period of the crescent design^34^. Figure 19b shows that the c-Si substrate can etched to a specified depth. For the axial junction, the traditional phosphorous diffusion process at 850 $^\circ$C is utilized using as the dopant source, phosphorous oxychoride^50^. The optimum thickness of the layer can be controlled by monitoring the temperature and duration of the diffusion process^50^. For the core–shell junction, the hot-wire chemical vapor deposition (HWCVD) technique can be employed for shallow-doping of the NW shell, used in the width range of 10 nm^50^. In this method, low temperature phosphorus doping, with a doping gas, is used at a substrate temperature of 250 $^\circ$C. The doping gas was $\hbox {H}_2$-diluted $\hbox {PH}_3$ (0.5%), with a pressure and a flow rate of 2 Pa and 20 standard cubic centimetres per minute (sccm), respectively^50^. After formation of the n/p/$\hbox {p}^+$ junction, a 200 nm thick ITO can be grown by the use of sputter-deposition for the top electrode^56^. To protect the NW and ITO layers from damage during the deposition of the bottom metal contact, a thick photoresist layer is deposited on the top surface. A diluted HF solution is used to remove the base $\hbox {SiO}_2$ layer from the wafer. Finally, metallic Ag (200 nm in thickness) could be deposited on the back of the substrate^25^. Finally, the QCr-NW proposed was annealed for 10 min at 400 $^\circ$C to decrease the surface recombination at the Ag-BSF layer interface^25^. Figure 19 shows these fabrication steps proposed in this work to obtain the axial and core–shell junctions.

## Conclusion

In this work, a quad-crescent-shaped silicon nanowire (NW) approach has been presented to enhance the light trapping performance of the thin film solar cells discussed. Here, the electrical performance of the cells has been investigated, taking into consideration the carrier lifetime and all the recombination processes. The PCE of the NW proposed in the axial and core–shell junctions was equal to 18.5% and 19%, with an enhancement of 23%, compared to 15% and 15.4% for the conventional NW design, respectively. These enhancements arose from the lateral geometry of the design proposed that increased the multiple scattering of the light between the NWs. Further, the significant improvement of the design proposed in the core–shell junction arose from the smaller lateral path length of the carriers with better collection efficiency. Therefore, the core–shell junction plays a significant impact on the performance of the NW design proposed. For each of the axial and core–shell junctions, the optoelectronic performance has been slightly affected by the top axial shell, up to a 300-nm thickness. However, the radial shell should be kept thin to improve the junction performance. Consequently, the core–shell junction of the design proposed offers better electrical performance and a lower influence of the surface recombination, compared to the axial junction.
